# Assessing customized multivalent chemokine-binding peptide treatment in a murine model of coxsackievirus B3 myocarditis

**DOI:** 10.1007/s00395-025-01098-w

**Published:** 2025-02-26

**Authors:** Nicolas Kelm, Meike Kespohl, Gintare Smagurauskaite, Serena Vales, Kalimuthu Karuppanan, Philomena Mburu, Arne Thiele, Sandra Pinkert, Thomas Bukur, Michael Mülleder, Nikolaus Berndt, Karin Klingel, Matthias M. Gaida, Shoumo Bhattacharya, Antje Beling

**Affiliations:** 1 Institute of Biochemistry, Charité, Universitätsmedizin Berlin, Corporate Member of Freie Universität Berlin and Humboldt-Universität zu Berlin, 10117 Berlin, Germany; 2 Deutsches Zentrum Für Herz-Kreislauf-Forschung, Partner Site Berlin, 10117 Berlin, Germany; 3 Centre for Human Genetics and RDM Cardiovascular Medicine, University of Oxford, Roosevelt Drive, Oxford, OX3 7BN UK; 4 Max Rubner Center for Cardiovascular Metabolic Renal Research, Institute of Pharmacology, Charité - Universitätsmedizin Berlin, Berlin, Germany; 5 Experimental and Clinical Research Center, A Joint Cooperation of Max-Delbrück Center for Molecular Medicine and Charité, Universitätsmedizin Berlin, Berlin, Germany; 6 Department of Nephrology and Intensive Care Medicine, Charité - Universitätsmedizin Berlin, Berlin, Germany; 7 Max-Delbrück-Center for Molecular Medicine in the Helmholtz Association, Berlin, Germany; 8 TRON, Translational Oncology at the University Medical Center of the Johannes Gutenberg University Mainz, Mainz, Germany; 9 Core Facility High-Throughput Mass Spectrometry, Charité-Universitätsmedizin Berlin, Freie Universität Berlin and Humboldt-Universität zu Berlin, Berlin, Germany; 10 Deutsches Herzzentrum der Charité, Institute of Computer-Assisted Cardiovascular Medicine, Berlin, Germany; 11 Charité-Universitätsmedizin Berlin, Corporate Member of Freie Universität Berlin and Humboldt-Universität zu Berlin, Berlin, Germany; 12 Department of Molecular Toxicology, German Institute of Human Nutrition Potsdam-Rehbruecke, Nuthetal, Germany; 13 Institute for Pathology and Neuropathology, University Hospital Tübingen, 72076 Tübingen, Germany; 14 Institute of Pathology, University Medical Center Mainz, Johannes-Gutenberg-Universität Mainz, 55131 Mainz, Germany; 15 Research Center for Immunotherapy, University Medical Center Mainz, Johannes-Gutenberg-Universität Mainz, 55131 Mainz, Germany

**Keywords:** Infection, Inflammation, Chemokines, Evasin, Myocarditis, Heart failure

## Abstract

Myocarditis, an inflammatory disease of the heart muscle, is often triggered by viral infections. This inflammation, which can lead to severe cardiac dysfunction and adverse outcomes, is mediated by various CC and CXC chemokines that interact with receptors in a “one-to-many” fashion. Ticks have evolved chemokine-binding salivary proteins known as Evasins, which efficiently suppress inflammation. This study explores a tailored Evasin-derived CC chemokine-targeting strategy using a 17-mer synthetic dimeric peptide, BK1.3. This peptide inhibits the inflammatory chemokines CCL2, CCL3, CCL7, and CCL8 in murine Coxsackievirus B3 (CVB3) infection, a viral trigger of myocarditis. Administered at a dose of 5 mg/kg twice daily, BK1.3 effectively maintains virus control without exacerbating CVB3-induced morbidity markers, such as hemodynamic compromise, multiorgan failure with hepatitis and pancreatitis, hypothermia, hypoglycemia, and weight loss. Metabolic profiling combined with proteomics reveals preserved reprogramming of lipid storage and gluconeogenesis capacity in the liver, alongside sustained energy production in the injured heart muscle. In survivors of acute CVB3 infection exhibiting manifestations of the subacute phase, BK1.3 enhances virus control, reduces myeloid cell infiltration in the heart and liver, improves markers of liver injury, and alleviates cardiac dysfunction, as evidenced by echocardiographic global longitudinal strain analysis. These findings affirm the safety profile of BK1.3 peptide therapeutics in a preclinical mouse model of acute CVB3 infection and emphasize its potential for therapeutic advancement in addressing virus-induced inflammation in the heart.

**Supplementary Information:**

The online version contains supplementary material available at 10.1007/s00395-025-01098-w.

## Introduction

Myocarditis is defined as an inflammatory disease of the myocardium, which is most commonly caused by viral infections, but also by other etiologies such as bacterial or protozoal infection, autoimmunity, drugs and toxins [17, 53]. Patients may present with severe clinical symptoms of acute heart failure or even suffer sudden cardiac death [2]. Myocarditis resolves in most patients spontaneously, however in some cases it persists and can lead to dilated cardiomyopathy [4, 18, 60]. Excessive immune responses triggered by the underlying causative event have been identified as a major contributor to cardiac damage. As such, in viral myocarditis, the immune response induced to combat the pathogen may cause substantial inflammatory tissue damage. This etiology is commonly referred to as immunopathology, which aggravates cardiac cell death and initiates fibrosis [36]. In line with this, immune cell infiltration into the myocardium is an independent predictor of cardiac death in patients with suspected myocarditis [37]. Likewise, immunosuppressive or immunomodulatory therapies are promising approaches that have partially been investigated in clinical trials [17, 19]. However, there is a lack of customized, targeted therapeutics that precisely tune the infiltration of specific immune cells known to have detrimental effects on cardiac homeostasis. Ongoing research aims to identify compounds with such a narrow activity spectrum.

Endomyocardial biopsy (EMB) is not only an important diagnostic procedure in patients with myocarditis but also provides important clues for potential druggable molecular targets [55]. The chemokine network emerged as such a therapeutic target in myocarditis [47]. Chemokines are released in response to infection-trigged tissue damage and attract circulating immune cells with matching receptor profiles; binding to G-protein-coupled chemokine receptors on these cells leads to their recruitment to the inflamed tissue. In patients with autoimmune myocarditis elevated levels of the chemokines *CCL20* [42], *CCL5* [38], *CCL13* [38], *CCL18* [38], *CCL2* [28, 29, 44], *CXCL9* and CXCL16 [12] are observed in the heart. Infiltrating immune cells can produce chemokines themselves and this results in amplified feed-forward loops with potentially detrimental effects [13]. As of today, approximately 50 different chemokines, which are grouped into CC-, CXC-, CX3C- and XC-chemokines in relation to the N-terminal spacing of their cysteine residues, and 20 chemokine receptors have been discovered in humans [32]. The upregulation of numerous chemokines and the one-to-many fashion of the chemokine-chemokine receptor interaction—where one chemokine can bind multiple receptors and *vice* versa,—make the chemokine network extraordinary robust and difficult to overcome [9]. Consequently, attempts to target the chemokine network with monovalent anti-chemokine agents have been unsuccessful so far [59]. However, targeted and customized inhibition of several disease-relevant chemokines could be a promising approach.

Blood-feeding ticks have evolved a mechanism to disrupt chemokine-driven inflammation with secreted salivary gland proteins, so-called Evasins [11]. Evasins can bind several chemokines with high affinity, interfering with the chemokine-chemokine receptor interaction [25] and inhibiting chemokine receptor signaling. The bioinformatic search in salivary gland transcriptome databanks and subsequent screening using yeast-surface display led to the discovery of over 50 distinct Evasins in tick species [1, 27, 43, 57]. Evasins are classified into class A and class B based on their exclusive binding properties to CC or CXC chemokines, respectively [10]. Importantly, Evasins within the same class can still exhibit distinct binding affinities for different chemokines, resulting in a unique binding spectrum for each. This diversity of chemokine-binding Evasins could be harnessed therapeutically by aligning their binding spectra with the chemokine expression profiles of inflammatory diseases [11]. Preclinical studies have demonstrated the anti-inflammatory potential of Evasins, including their effectiveness in cardiac inflammation related to ischemic heart disease [14, 48]. Despite their promise, intact Evasin protein therapeutics face challenges such as restricted oral bioavailability, high production costs, and immunogenicity. In contrast, peptide therapeutics generally offer lower manufacturing costs and improved feasibility for targeted treatments. For instance, the class A Evasin P672, which neutralizes several CC-chemokines with high affinity [27], led to the development of the BK1.3 peptide—a 17-mer dimeric peptide with an intramolecular disulfide bond, demonstrating effective inhibition of CCL2, CCL3, CCL7, and CCL8 [23].

To explore the potential of such peptide-based therapies to mitigate cardiac inflammation, we aimed to evaluate the BK1.3 peptide in a preclinical model of viral myocarditis. Coxsackievirus B3 (CVB3) infection in mice represents an ideal model for this purpose [22]. Intraperitoneal injection of this cardiotropic enterovirus causes systemic virus spread and cardiomyocyte infection [52]. The resulting presence of pathogen-associated molecular patterns (PAMPs) and increased release of damage-associated molecular patterns (DAMPs) due to viral cytotoxicity trigger a local immune response with cytokine and chemokine release. This response leads to the infiltration of predominantly myeloid immune cells into cardiac tissue [3, 47, 52], mimicking human disease [3, 45, 47]. Notable chemokines, such as *Ccl2* [3], *Ccl3* [21, 30] and *Ccl7* [50], are significantly upregulated in CVB3-induced murine myocarditis. Given that the multivalent chemokine-binding peptide BK1.3 targets these disease-relevant chemokines, we aimed to assess its efficacy in reducing cardiac inflammation in a murine CVB3 myocarditis model.

As with any drug development, the safety of BK1.3 during CVB3-induced myocarditis must be carefully evaluated. While targeting the immunopathology of CVB3-induced myocarditis, it is crucial to maintain virus control. Myocarditis in mice follows an acute phase of CVB3 infection characterized by severe damage to the pancreas and liver, systemic deterioration and hypodynamic circulation. Immune mechanisms play a critical role in preventing exacerbation of the viral infection during this phase [40, 63]. Hepatocytes trap viral particles from the blood, albeit at the cost of hepatic necrosis, which resolves with minor residual injury by the myocarditis stage [34, 35]. With *Ccl2* [33] upregulation being observed in the liver, the role of the chemokine network during acute CVB3 infection remains unclear for the restoration of liver function. Additionally, the acute phase with increased energy expenditure and lower availability of plasma nutrients requires metabolic adjustments in liver [34] and heart muscle [15]. Therefore, this study assessed not only the impact of BK1.3 on cardiac inflammation but also thoroughly investigated its effects on other organ manifestations to ensure a comprehensive safety profile.

## Material and methods

### Peptide synthesis of BK1.3 and BK1.3 SCR dimers via disulfide bond formation

The identification of P672 and BK1.3, their structure, chemokine-binding affinities and anti-inflammatory properties have been described elsewhere [23, 27]. Peptides were synthesized by the peptide synthesis facility at Charité Universitätsmedizin Berlin. The peptides BK1.3 (NH_2_-YEDEDYEDFFKPVTCYF-COOH) and its scrambled variant BK1.3 SCR (NH_2_-VEYFYDYDFEKFCEPTD-COOH) were synthesized as dimers through disulfide bond formation using an automated microwave-assisted peptide synthesizer Liberty Blue (CEM, Matthews, NC, USA). The synthesis was conducted on Rink Amide ProTide Resin with 0.19 mmol/g loading (CEM), employing standard Fmoc chemistry. Post-synthesis, peptides were cleaved from the resin with a trifluoroacetic acid-based cocktail (Thermo Fisher Scientific, Waltham, MA, USA), precipitated with diethyl ether (Carl Roth, Karlsruhe, Germany), dissolved using 10% acetic acid (Carl Roth) and lyophilized. Crude peptides were purified using preparative HPLC (Shimadzu Germany, Duisburg, Germany) on a C18 column (Kromasil, Bohus, Sweden) with gradient elution, followed by lyophilization to yield uniform batches. The formation of dimers was achieved by adjusting the pH of the peptide solution to 8.5–9.0 and incubating it with activated carbon (VWR, Radnor, PA, USA) at room temperature, monitored via analytical HPLC (Shimadzu Germany) on a Zobrax C18 column (Agilent Technologies, Santa Clara, CA, USA) to confirm the shift in retention time indicative of dimerization. Final products were characterized by HPLC (Shimadzu Germany) and MALDI-TOF mass spectrometry (Bruker, Billerica, MA, USA), confirming the successful synthesis and purity of both BK1.3 and BK1.3 SCR dimers. Quality control assessment of BK1.3 and its scrambled variant SCR included a THP-1 cell migration assay, following methods described previously [61]. The assays demonstrated an IC_50_ of 4.2 nM for BK1.3 against CCL8-induced cell migration, which is consistent with previous reports [23]. BK1.3 and SCR were stored as lyophilized powders. Before injection, peptides were thawed and dissolved in 0.5% DMSO (Biomol, Hamburg, Germany). The volume administered per intraperitoneal injection was 100 µL. The dosage of 5 mg/kg body weight was based on the average baseline weight of the mice.

### BK1.3 quality testing in acute LPS-induced lung inflammation mouse model

To study the antiinflammatory efficacy of the produced BK1.3 batch in vivo, we employed an established model of acute LPS-induced lung inflammation [46]. Animal procedures were approved and carried out in accordance with the United Kingdom Home Office Animals (Scientific Procedures) Act 1986, under project licence PPLP973A60F5. In summary, 8–10-week-old male C57BL/6 J mice (Charles River, United Kingdom) were treated with 2 mg/kg of LPS (Lipopolysaccharides, L6143; Merck, Darmstadt, Germany) in a final volume of 20 µL PBS (Gibco, Thermo Fisher Scientific) intranasally. For this procedure, mice were narcotized with 3.5% isoflurane (Zoetis, Parsippany, NJ, USA) and the whole volume of LPS solution was slowly distributed between both nostrils. At the time of lung inflammation induction, mice received an intraperitoneal injection of either BK1.3 (5 mg/kg), SCR (5 mg/kg) or PBS in a final injection volume of 100 µL. A second intraperitoneal dose of BK1.3, SCR or PBS was repeated 9 h later. Mice were sacrificed by exsanguination under terminal anaesthesia 24 h after induction of lung inflammation. Tracheal intubation for bronchoalveolar lavage fluid (BALF) collection was performed post-mortem by washing the lung twice with 1 mL of PBS using a 2.5 mL syringe fixed to a 22G Vasofix safety catheter (VWR).

Immediately after collection, BALFs were centrifuged for 10 min at 500 g 4 °C, the supernatant was removed, and cells resuspended in fetal calf serum-containing (FCS) stain buffer (BD, Franklin Lakes, NJ, USA). Mouse Fc Block (BD) was applied in a 1:200 dilution for 30 min at 4 °C, the samples were washed with stain buffer and subsequently staining was performed with an antibody mix for 30 min in the dark at 4 °C. The antibody-fluorochrome conjugates included in the antibody mix were provided by Miltenyi (Bergisch Gladbach, Germany). More details on the antibody-fluorochrome conjugates are given in Supplemental Table S1. After another wash step, samples were fixed in 2% formaldehyde (Thermo Fisher Scientific) for 10 min and 1 µL of Fixable dead stain near-IR (Thermo Fisher Scientific) was added to each sample. Measurements were immediately done on the Attune Flow Cytometer (Thermo Fisher Scientific). Fluorophore spill-over was compensated carefully. Attune™ Cytometric Software v5.2.0 (Thermo Fisher Scientific) and FlowJo V10.6.2 software (Ashland, Wilmington, DE, USA) were used for analysis. A representative image of the gating strategy is depicted in Fig. 2C.

### Production of Evasin P672

The P672 protein was produced as a secreted N-terminal His8-StrepII tagged protein in mammalian cells as described [27]. HEK293F cells were transiently transfected with a plasmid containing the P672 sequence and an N-terminal His8-StrepII purification tag (HHHHHHHHSAWSHPQFEKGGGGS). P672 was purified from the cell culture supernatant using nickel affinity chromatography, followed by size exclusion chromatography to ensure high purity. The purity, molecular weight, and glycosylation status of the protein were confirmed by SDS-PAGE, with results consistent with published data [27]. To assess the biological activity of P672, an in vitro CCL8-induced THP1 cell migration assay was performed. The assay measured the ability of P672 to inhibit chemotaxis, with IC_50_ values determined to be in the range of 4.6 to 10.0 nM. The purified P672 protein was stored in PBS (Thermo Fisher Scientific) at −80 °C until use. Before injection, the protein was thawed and diluted in 0.5% DMSO to prepare the injection solution. For intraperitoneal administration, 100 µL of the protein solution was injected into each subject at a dosage of 5 mg/kg body weight, based on the average baseline weight of the mice.

### Animals

6- to 8-week-old male C57BL/6 J mice were purchased from Charles River, Germany or provided by the breeding facility of Charité—Universitätsmedizin Berlin Charité. Littermates from each cage were randomly assigned to the treatment and control group. Mice were intraperitoneally infected with 10^5^ plaque-forming units of CVB3-H3. The virus batch was produced by transfection of CVB3-H3 cDNA (pBKCMV-H3) into HEK293T cells and amplified in HeLa cells as described elsewhere [39]. Intraperitoneal injection of Evasin peptide BK1.3 (5 mg/kg body weight) or the control peptide SCR (5 mg/kg body weight) was initiated 12 h after infection and continued at 12-h intervals until the end of the experiment. Body weight was measured at least once a day and rectal body temperature was taken at baseline, on day 3 and on day 8 post infection. The animals were euthanized using a lethal overdose of isoflurane (CP-Pharma, Burgdorf, Germany) on days 3 and 8 post-infection for subsequent analysis. Retrobulbar blood was collected immediately before euthanisation at the aforementioned time points and subsequently blood glucose levels were measured with Accu-Chek glucometer (Roche Diabetes Care, Indianapolis, IN, USA). Afterwards, systemic circulation was flushed by PBS (AppliChem, Darmstadt, Germany) injection into the heart and the mice were dissected. The removed organs were weighed, directly frozen in liquid nitrogen and later stored at −80 °C.

All animal experiments conducted in this study were approved by the local animal welfare authorities in Berlin (“Landesamt für Gesundheit und Soziales”), registered under permit number G0139/20. We adhered strictly to the German Animal Welfare Act and the Animal Welfare Laboratory Animal Regulations, in accordance with the European Directive 2010/63/EU on the protection of animals used for scientific purposes. Measures to minimize suffering were rigorously implemented. Oral administration of Tramadol (Grünenthal, Stolberg, Germany) via drinking water was used for refinement, following our previously published protocol [52]. Animal welfare was assessed at least twice daily and recorded in score sheets that included parameters such as body weight, general condition, breathing pattern, behavior, and signs of pain. Animals meeting termination criteria as per the score sheets were euthanized to prevent undue suffering.

### Histology and immunohistochemistry

Tissue samples were taken from the heart, pancreas and liver and fixed in 4% formaldehyde solution (Carl Roth). The fixed samples were stored refrigerated in PBS (AppliChem) until they were embedded in paraffin, sectioned and stained. Hematoxylin and eosin (H&E) staining was carried out for all organs. The cardiac inflammation was assessed by the blinded rating of the myocarditis score (0: no inflammatory infiltrates, 1: small foci of inflammatory cells between myocytes, 2: larger foci of > 100 inflammatory cells, 3: ≤ 10% of cross-section involved, 4: 10%–30% of a cross-section involved) [58]. Pancreas destruction was evaluated with a scoring system ranging from 0 to 100% destruction. Following a histological scoring system used by Veteläinen et al. [62], liver tissue sections were graded for inflammation and necrosis (0: no inflammation/necrosis; 1: scattered immune cells/mild necrosis (< 10%), 2: immune cell foci/marked necrosis (10%–50%), and 3: diffuse immune cell infiltrates/severe necrosis (> 50%)). F4/80 immune cells were identified by staining with an F4/80 antibody (BM8, 14–4801-85, Invitrogen) and nucleated F4/80^+^ cells were counted in high-power fields.

### Serum analysis

Whole blood obtained from retrobulbar sampling was centrifuged at 10,000 × g for 10 min. The serum supernatant was separated and stored at −80 °C. Serum levels of albumin and enzyme activities of aspartate aminotransferase (ASAT), alanine aminotransferase (ALAT) and alkaline phosphatase (AP) were measured by the veterinary diagnostic laboratory (SYNLAB Vet, Berlin, Germany). Free fatty acid serum levels were determined with a colorimetric Free Fatty Acid Kit (Abcam, Waltham, MA, USA) in accordance with the manufacturer’s instructions.

### Echocardiography

Baseline echocardiography was performed on all mice two days prior to infection. During the infection, echocardiography was conducted on day 3 for animals sacrificed on that day and on day 7 for those sacrificed on day 8. The echocardiography was carried out using the Vevo 3100 echocardiography machine (FUJIFILM VisualSonics, Toronto, ON, Canada), an MX400 ultrasound probe, a heated examination table with electrodes, heated ultrasound gel (Parker Laboratories, Fairfield, NJ, USA), and a heat lamp. Anesthesia in mice was induced with 3–5% isoflurane (CP-Pharma) inside an anesthesia chamber and was continued with 1.5–2% isoflurane maintenance dose via non-invasive mask ventilation during the echocardiography session. Heart rate, respiratory rate, and body temperature were continuously monitored and kept within physiological ranges throughout the examination. All measurements were performed by a single, experienced investigator.

Echocardiographic analysis included image acquisition of the parasternal long-axis, mid-papillary parasternal short-axis, and apical 4-chamber views, as well as pulsed-wave Doppler of mitral blood flow and tissue Doppler of the septal mitral annulus. Measurements adhered to small animal echocardiography guidelines [64]. Post-acquisition image analysis was conducted using VevoLab 3.2.1 software (FUJIFILM VisualSonics), with each outcome parameter averaged from data obtained from at least three image analyses.

End-diastolic and end-systolic left ventricular volumes, stroke volume, cardiac output, and ejection fraction were derived from the parasternal long-axis B-mode view using the Simpson method. Left ventricular anterior and posterior wall thicknesses during systole and diastole were measured from M-mode images acquired in the parasternal short-axis view. Blood flow velocities for early (E) and atrial (A) diastolic filling were measured with transmitral pulsed-wave Doppler. Isovolumetric relaxation and contraction times, as well as aortic ejection time, were quantified. Tissue Doppler imaging of the septal mitral annulus was used to determine wall motion velocities during early (E’), atrial (A’), and systolic (S’) phases. Additionally, speckle tracking analysis was performed using VevoLab 3.2.1 software. Guidelines on speckle-tracking in mice were considered [5]. Endocardial and epicardial borders were traced over three ECG-monitored respiratory artifact-free heart cycles in a parasternal long-axis view. Global longitudinal strain was derived from at least three separate image acquisitions and the average was calculated.

### Immune cell isolation from organs

#### Splenocytes

The protocol for splenocyte isolation is described elsewhere [52].

#### Heart tissue

Removed heart tissue was weighed and minced into small pieces in cooled, supplemented RPMI medium (Life Technologies, Waltham, MA, USA) containing 2% fetal calf serum (FCS; Sigma-Aldrich, St. Louis, MO, USA), 30 mM HEPES (Life Technologies) and 1% penicillin/streptomycin (Life Technologies). Collagenase 2 (Worthington, Lakewood, NJ, USA) and DNase I (Sigma-Aldrich) were added to a final concentration of 1 mg/ml and 0.15 mg/ml, respectively, and the tissue was incubated for 30 min at 37 °C with continuous shaking. Enzymatic digestion was halted with 10 mM EDTA (Ethylenediaminetetraacetic acid, VWR), and the tissue solution was filtered through a 70 µm cell strainer (Corning, Corning, NJ, USA). The samples were then centrifuged at 310 × g for 10 min at 4 °C, and the supernatants were carefully removed. Red blood cell lysis was performed using a buffer (10 mM KHCO_3_ (Carl Roth), 155 mM NH_4_Cl (Thermo Fisher Scientific), 0.1 mM EDTA (VWR) in distilled water) for 3 min at room temperature. After centrifugation, the leukocyte pellets were resuspended in FACS buffer containing 2% FCS (Sigma-Aldrich) and 2 mM EDTA.

#### Hepatic tissue

Liver pieces were weighed and placed in a supplemented RPMI medium containing 0.1% bovine serum albumin (BSA; AppliChem) and 1% penicillin/streptomycin (Life Technologies) on ice. The liver tissue was cut into small pieces, and Collagenase 4 (Worthington) was added to a final concentration of 0.2%. The samples were incubated for 20 min at 37 °C, followed by the addition of DNase I to a final concentration of 0.9 mg/ml and further incubation under the same conditions. Digestion was stopped with 2 mM EDTA and 0.1% BSA in HBSS (Hanks’ Balanced Salt Solution; Thermo Fisher Scientific), and the tissue was filtered through a 70 µm cell strainer. The tissue solution was then diluted with cooled PBS and centrifuged twice at 55 × g for 1 min at 4 °C, discarding the pellet each time. A final centrifugation was performed at 500 × g for 10 min at 4 °C, and the supernatant was removed. The pellet was resuspended in 6 ml of 30% Nycodenz solution (Serumwerk Bernburg, Bernburg, Germany), and 4 ml of HBSS containing 0.1% BSA was added. Additional 6 ml of HBSS with 0.1% BSA were carefully layered on top to create two distinct phases. Gradient centrifugation was carried out for 22 min at 4 °C and 1,400 × g with slow acceleration and no deceleration, producing a white mid-layer containing leukocyte populations. The leukocytes were extracted by pipetting, pelleted by centrifugation at 500 × g for 10 min at 4 °C, and resuspended in FACS buffer. Leukocyte concentration was determined using a Neubauer counting chamber (Hirschmann Laborgeräte, Eberstadt, Germany).

### Flow cytometry

Immune cells isolated from 20 mg of heart tissue and a specified number of cells from spleen and liver tissues were used for flow cytometry staining. Samples were first incubated with an Fc blocking agent (Miltenyi) at a 1:50 dilution for 20 min at 4 °C. This was followed by staining with an antibody mix for 20 min at 4 °C in the dark. The used antibody-fluorochrome conjugates were provided by BD, Biolegend (San Diego, CA, USA) and Life Technologies. More details on the antibody-fluorochrome conjugates are given in Supplemental Table S1. Samples were then washed with PBS and centrifuged for 3 min at 4 °C and 300 × g. Live/dead staining was performed with Fixable Viability Dye eFluor 780 (eBioscience, San Diego, CA, USA), incubated at a 1:1000 dilution in PBS for 30 min in the dark. After another wash, samples were fixed with 2% formaldehyde (Carl Roth), then washed and resuspended in FACS buffer. For absolute immune cell counts, 123 count eBeads (Life Technologies) were added to heart samples. Flow cytometry was conducted using a FACSymphony instrument (BD), and data were analyzed with FlowJo V10.6.2 software. Representative gating strategies are shown in Supplemental Figs. S1, S2, S3.

### Quantification of infectious viral particles

Virus titer was quantified by plaque assay. HeLa cells (Henrietta Lacks-derived; ATCC, Manassas, VA, USA) were cultured in supplemented MEM (Life Technologies) containing 5% FCS (Sigma-Aldrich), 1% penicillin/streptomycin (Life Technologies), 2% HEPES (Life Technologies), and 1% non-essential amino acids (NEAA) (Life Technologies). HeLa monolayers were seeded in 24-well plates (Greiner, Kremsmünster, Austria). Tissue samples from the heart, liver, spleen, and pancreas were weighed and homogenized in 1 ml of MEM using 2 mm Zirconia beads (BioSpec Products, Bartlesville, OK, USA) in a FastPrep machine (Savant Instruments, Hyderabad, Telangana, India). Serial tenfold dilutions of the tissue suspensions were applied to the HeLa monolayers and incubated for 30 min at 37 °C. Following incubation, the supernatants were removed, and the infected monolayers were overlaid with Eagle’s agar containing MEM, 0.68% penicillin/streptomycin, 1.6 g/l NaHCO_3_ (Carl Roth), 9% FCS, and 0.7% Difco Agar Noble (BD Bioscience, Heidelberg, Germany). After two days, cell lysis plaques were stained with MTT (3-(4,5-dimethylthiazol-2-yl)−2,5-diphenyltetrazolium bromide; Sigma-Aldrich) and counted. For each tenfold dilution, two replicates were analyzed, and results were expressed as plaque-forming units (pfu) per gram organ tissue.

### Quantitative Real-Time PCR

Liver tissue was homogenized, and RNA was extracted using Trizol (Thermo Fisher Scientific) following the manufacturer’s instructions. The final RNA concentration was determined using a NanoDrop spectrophotometer (VWR). For cDNA synthesis and subsequent quantitative real-time PCR, Qiagen’s 2-Step RT-PCR 96-well protocol was strictly followed. A total of 2.5 µg of RNA was used for cDNA synthesis with the QuantiNova Reverse Transcription Kit (Qiagen, Venlo, Netherlands). The synthesized cDNA was then mixed with the QuantiNova SYBR Green PCR Kit (Qiagen) and applied to the PCR array of the Mouse Chemokines & Receptors QuantiNova® LNA® PCR Focus Panel (SBMM-022ZC; Qiagen). The PCR reactions were run on the StepOnePlus real-time PCR system (Thermo Fisher Scientific). Data analysis was conducted in an R environment using a library designed for high-throughput quantitative real-time PCR data [26]. CT values equal to or greater than 40 were excluded from further analysis. The CT values were scaled across all samples according to the ratio of the geometric means of each sample’s CT values. We performed differential gene expression analysis to determine ΔΔCT values between all pairs of experimental groups within each tissue. *Actb*, *Gapdh*, and *Hsp90ab1* were used as housekeeping genes. Statistical analyses were performed with the wrapper function limmaCtData (stringent = FALSE and spacing = 1) that employes the limma package [54] and its functions lmfit and ebayes with a contrast matrix providing empirical Bayes moderated t-statistics on the fitted linear models for each gene in all pairwise comparisons.

### Sample preparation for proteomics

Heart and liver tissues (5–20 mg) were weighed into Lysing Matrix D Tubes (MP Biomedicals, Irvine, CA, USA), and the volume was adjusted to 300 µl with RIPA lysis buffer (Thermo Fisher Scientific) supplemented with 1.25 × protease inhibitor (Merck). The samples were homogenized using a FastPrep device (MP Biomedicals) at 6 m/s for three cycles of 30 s each. After homogenization, debris was pelleted by centrifugation at 2,500 g for 5 min, and the protein concentration was determined using the Pierce Protein Assay Kit (Thermo Fisher Scientific). A total of 25 µg of protein was transferred into an Eppendorf TwinTec plate (Eppendorf, Hamburg, Germany) for SP3 protein preparation.

The lysates were processed on a Biomek i7 workstation (Beckman Coulter, Indianapolis, IN, USA) following the SP3 protocol, as previously described, with one-step reduction and alkylation [49]. Briefly, 16.6 μl of reduction and alkylation buffer (40 mM TCEP (tris(2-carboxyethyl)phosphine; Merck), 160 mM CAA (2-chloracetamide; Sigma-Alrdrich), 200 mM ABC (ammonium bicarbonate; Honeywell, Morris Plains, NJ, USA)) were added, and the samples were incubated at 95 °C for 5 min before cooling to room temperature. For protein binding, 250 μg of paramagnetic beads (in a 1:1 ratio of hydrophilic/hydrophobic beads; GE Healthcare Technologies, Chicago, IL, USA) were added, and proteins were precipitated with 50% acetonitrile (Thermo Fisher Scientific). The samples were washed twice with 80% ethanol (Sigma-Aldrich) and once with 100% acetonitrile before reconstitution in 35 μl of 100 mM ABC. The samples were digested overnight at 37 °C for 17 h using a Trypsin/LysC Mix stock solution at 0.1 µg/µl (Promega, Madison, WI, USA) at a protein ratio of 1:50 (w/w). The digestion was terminated by adding formic acid (Thermo Fisher Scientific) to a final concentration of 0.1%. Peptide concentration was measured using the Pierce Quantitative Fluorometric Peptide Assay (Thermo Fisher Scientific), and the samples were transferred to a new plate and stored at −80 °C until analysis by LC–MS/MS without further conditioning or clean-up.

### Proteome analysis by DIA LC–MS and DIA-NN

LC–MS analysis was conducted using an Evosep One system (Evosep Biosystems ApS, Odense, Denmark) coupled with a Bruker timsTOF Pro 1 mass spectrometer. A total of 200 ng of tryptic-digested peptides were loaded onto Evotip Pure tips (Evosep Biosystems ApS) following the manufacturer’s protocol. Liquid chromatography was performed using the Evosep 30 SPD LC method, which features a 44-min gradient, with an EV1137 performance column (ReproSil-Pur C18, 1.5 µm beads by Dr. Maisch, Ammerbuch, Germany, 15 cm × 150 µm) maintained at 50 °C. The column was coupled to a 10 µm Zero Dead Volume (ZDV) captive spray emitter. For mass spectrometry acquisition, the Bruker default method “dia-PASEF-long gradient” was employed. The acquisition covered a mass range of m/z 100 to 1700 and an ion mobility range of 1/K0 0.6—1.6, using 32 mass steps per cycle. The collision energy increased progressively depending on the ion mobility window, ranging from 20 to 59 eV, with a window width set to 50 Da. Accumulation and ramp times were maintained at 100 ms. Data processing steps of spectra deconvolution, protein identification and relative quantification were conducted using DIA-NN 1.8.1 (Data-Independent Acquisition by Neural Networks) [24]. The peptide search was performed in library-free mode, utilizing deep learning to create a new in silico spectral library from the Uniprot reference proteome from mus musculus (downloaded on August 27, 2023). DIA-NN standard settings were applied, precursor charge range was set to 2–4, scan window radius was set to 7, mass accuracy and MS1 accuracy set to 15, and match between runs activated.

### Computational simulation of liver metabolism in infection

We used HEPATOKIN1 [6] in combination with a detailed model of lipid droplet metabolism [7] to evaluate the functional implications of protein abundance changes observed during infection and treatment in the liver. The model comprises the central hepatic metabolic pathways of glycolysis, gluconeogenesis, glycogen synthesis, glycogenolysis, fructose metabolism, galactose metabolism, the creatine phosphate/ATP shuttle system, the pentose phosphate cycle composed of the oxidative and non-oxidative branch, the citric acid cycle, the malate aspartate redox shuttle, the glycerol-3-phosphate shuttle, the mitochondrial respiratory chain, the beta-oxidation of fatty acids, fatty acid synthesis, ketone body synthesis, cholesterol synthesis, TAG synthesis and degradation, the synthesis and hydrolysis of TAG, the synthesis and export of VLDL, the urea cycle, the metabolism of the amino acids serine, alanine, glutamate, glutamine, and aspartate, and ethanol metabolism. The model contains the key electrophysiological process of the inner mitochondrial membrane including the mitochondrial membrane potential, mitochondrial ion homeostasis, and the generation and utilization of the proton motive force described by kinetic equations of the Goldman-Hodgkin-Katz type [8]. We used the protein abundance data obtained from LC–MS/MS analysis to generate individual instantiations of the metabolic model describing the liver of each animal. For this purpose, the maximal activities ${V}_{max}$ of enzymes and transporters ${E}_{a}$ for each animal were computed according to ${v}_{\text{max}}^{{E}_{a}}={v}_{\text{max}}^{{E}_{ref}} \frac{{E}_{a}}{{E}_{ref}}$, where ${E}_{a}$ and ${E}_{ref}$ denote the label-free quantification intensities for protein E in the animal a and the reference liver, respectively. As a reference, we used the mean label-free quantification intensities of protein E in control livers. The maximal activity ${v}_{\text{max}}^{{E}_{ref}}$ of enzyme E for the reference livers was assumed to be identical to the generic model HEPATOKIN1 that has been fitted to experimental data of fluxes and metabolites obtained in perfused whole livers, isolated cells, or tissue sections derived from whole livers. All rate equations and the ${v}_{\text{max}}^{{E}_{ref}}$ values can be found in [6].

### Statistics

Statistical analysis was performed using GraphPad Prism V10.1.1 software (La Jolla, CA, USA) for Windows. Outliers in the primary data identified by the ROUT method (Q = 1%) were not considered for further statistical analysis and are not shown in the graphs. The D’Agostino-Pearson test was used to test the data for normal distribution. Unless otherwise indicated, individual data points, mean values, and standard deviations (SD) are depicted in each graph. Viral titer data were log10 transformed prior to statistical analysis. In the corresponding graphs, the log10 transformed data are plotted on a linear scale labelled to the power of 10. In general, the applied statistical test is indicated in the figure legends for each graph. In summary, two group comparisons were analyzed with unpaired *t*-tests (Figs. 2D, 7G, 8A-D, F-I, 10A-D, G). If an *F*-test determined unequal variances, an unpaired *t*-test with the Welch correction was performed. One-way ANOVA was used to compare three or more unmatched groups and was followed by multiple comparisons with either Dunnett´s or Tukey’s test (Figs. 3B, D-G, 4A-F, H-I, 5A-B, 6A-D, 7D-E). Data with the two factors of time and treatment were analyzed by the means of Two-way ANOVA with repeated measures in Fig. 7B and paired Two-way ANOVA test in Figs. 3C, 5C-D, 7F and 9C as well as Tables 1, 2. Two-way ANOVA test was followed by either Tukey or Sidak multiple comparisons as indicated in the figure legends. Survival rates were compared using the Log-rank (Mantel-Cox) test. Statistical analysis of proteomic data was performed by a two‐sample t‐test with Benjamini–Hochberg correction for multiple testing. Principal Component Analysis (PCA) was performed to reduce the dimensionality of the proteomic dataset. Using MATLAB (R2023b, MathWorks), the pca function was employed on the dataset. The results were used to identify the principal components that capture the most significant variance within the data. P-values of less than 0.05 were considered statistically significant in all analyses and are indicated by asterisks (*), non-significant results are indicated by “ns”. If the statistical results are not further specified within graphs, the corresponding statistical tests described in the figure legends were non-significant.

## Results

### Acute CVB3 infection induces upregulation of numerous chemokine network members

**Fig. 1:**
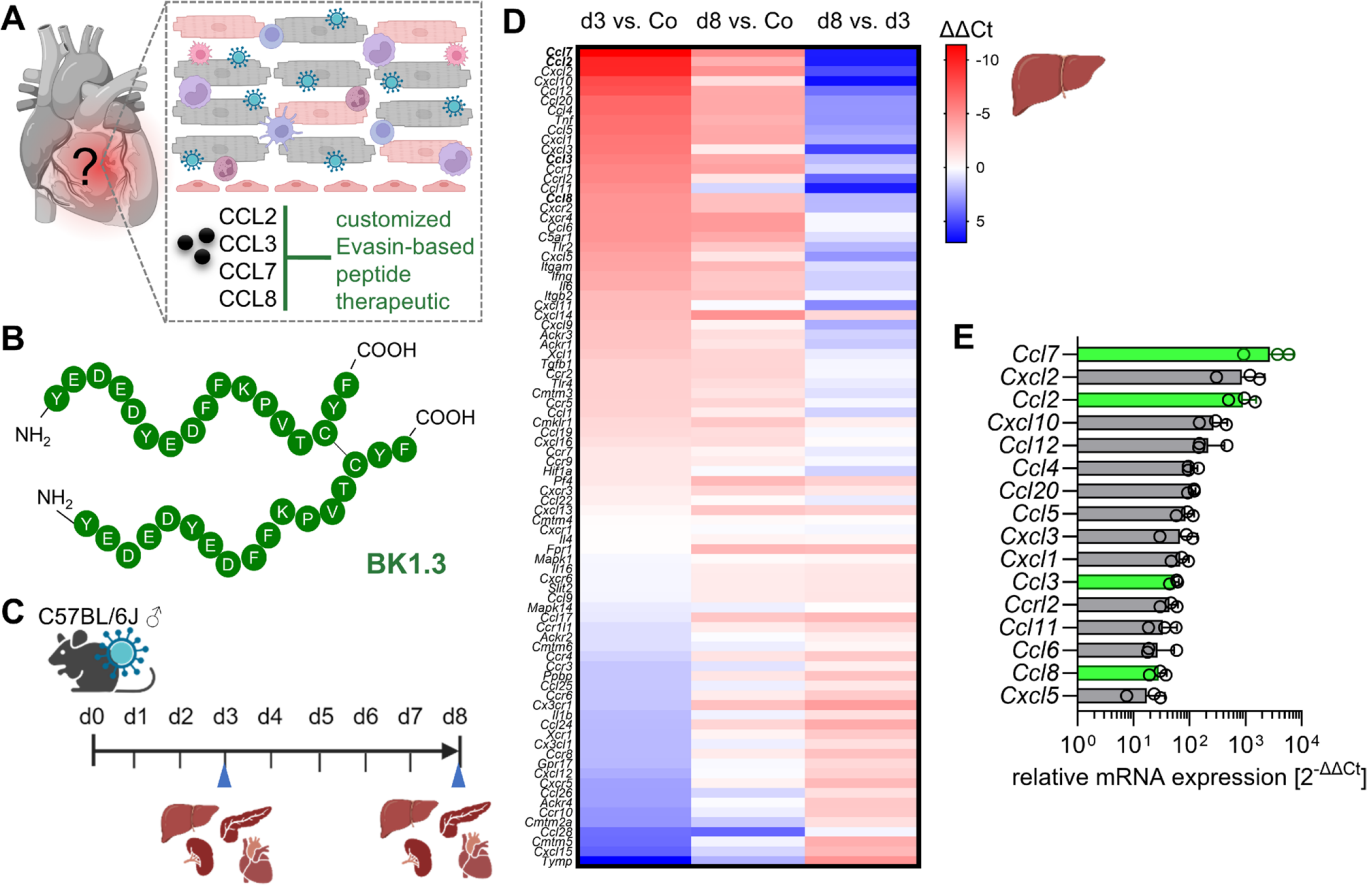
Characterization of chemokine profiles in a CVB3 infection mouse model. **A** Schematic representation of the anti-inflammatory strategy: Cardiac CVB3 infection triggers a chemokine network, leading to harmful immune cell infiltration. Targeting disease-specific chemokines with a custom Evasin-based peptide therapeutic (BK1.3) may reduce cardiac inflammation. **B** Amino acid sequence of the 17-mer synthetic dimeric peptide BK1.3. The sequence is displayed in white capital letters, with a black line indicating the disulfide bond between two cysteine residues. **C** Male C57BL/6 J mice were infected with CVB3 and euthanized on days 3 and 8. The illustrated organs were collected for analysis. **D** Chemokine profiling in liver tissue by RT-qPCR at baseline (co: control; *n* = 3), day 3 (*n* = 3), and day 8 (*n* = 3). The heatmap shows mean ΔΔCT values for the indicated chemokines, comparing day 3 vs. control (1st column), day 8 vs. control (2nd column), and day 8 vs. day 3 (3rd column). Red rectangles indicate upregulated mRNA expression, while blue rectangles denote downregulated mRNA expression. **E** Quantification of mRNA expression levels for chemokines in liver tissue was performed on day 3, relative to baseline levels in control mice. Chemokines targeted by BK1.3 are highlighted in green. Data are presented as mean ± SD. Significantly upregulated chemokines were identified through pairwise comparisons across all groups, using the limma package and the function limmaCtData in the R environment

Our study focuses on developing Evasin-derived peptide therapeutics to treat viral myocarditis. Based on existing research, we have identified CCL2, CCL3, CCL7, and CCL8 as primary targets for anti-inflammatory intervention in viral myocarditis (Fig. 1A). As a result, we chose to explore the BK1.3 dimerized peptide, derived from the P672 Evasin, which demonstrates potent inhibition of these chemokines (Fig. 1B). To evaluate the therapeutic potential of BK1.3, we infected C57BL/6 J mice with the cardiotropic virus CVB3 (H3) and assessed myocarditis severity, which typically peaks around days 7 and 8 post-infection. The mouse model we employed exhibits early systemic involvement, making it suitable for assessing BK1.3's efficacy in reducing cardiac inflammation even prior to and during the onset of myocardial tissue damage (Fig. 1C). We hypothesized that BK1.3 might also reduce inflammation in other organs, such as the liver, which experiences significant damage during early infection stages. Consequently, we investigated the chemokine response and inflammation during the early (day 3), but also for late (day 7/8) phases of CVB3 infection in both the heart and liver. Given that chemokine network inhibition with BK1.3 might interfere with virus control, it is crucial to consider the safety of BK1.3 during all stages of CVB3 infection, including the acute phase on day 3, characterized by acute pancreatitis and hepatitis, as well as organ involvement during the subacute myocarditis stage on day 8 (Fig. 1C). Therefore, our assessment of BK1.3 thoroughly characterizes all aspects of murine CVB3 infection.

Given that CVB3 most severely impacts the liver during the early late stages and a detailed analysis of the entire chemokine network was lacking, we prioritized a comprehensive evaluation of the chemokine network across different infection phases for this organ. We performed mRNA gene expression profiling of the chemokine network on liver tissues at days 3 and 8 post-infection, comparing the results with uninfected controls. Our findings revealed a broad and robust induction of chemokines, particularly on day 3, which marks the peak of acute inflammation and necrosis in the liver [34]. Notably, chemokines such as *Ccl7* (3,760-fold), *Cxcl2* (1,202-fold), and *Ccl2* (958-fold) (Fig. 1D/E), which are crucial for recruiting monocytes and neutrophils to the infection site, were significantly upregulated. By day 8, although chemokine expression levels remained elevated compared to controls, they showed a noticeable decline, indicating the onset of inflammation resolution (Fig. 1D). Importantly, the chemokines targeted by BK1.3—CCL2, CCL3, CCL7, and CCL8—were among those significantly upregulated in the liver during the acute phase (Fig. 1E), corroborating their involvement in the inflammatory response observed in CVB3-infected cardiac tissue. This widespread chemokine induction supports targeting the chemokine network with a multivalent inhibitor like BK1.3, but it also underscores the necessity of vigilant monitoring of non-cardiac organ manifestations to detect potential adverse effects from systemic chemokine inhibition.

### BK1.3 peptide synthesis yields a potent inhibitor of chemokine-mediated cell migration

**Fig. 2:**
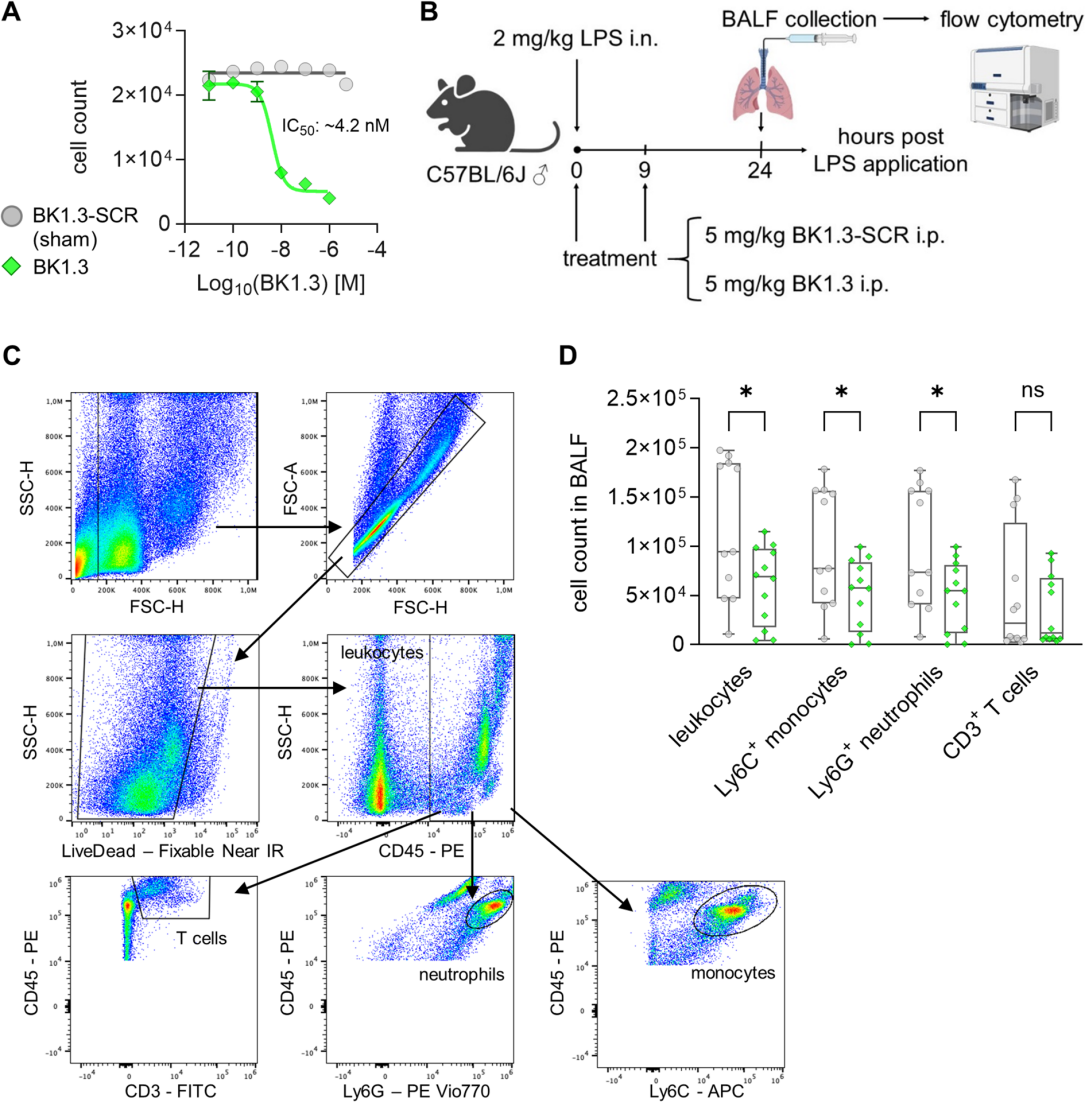
Validation of chemokine-mediated chemotaxis inhibition by BK1.3. **A** A cell migration assay was conducted using THP-1 cells, with CCL8-induced migration to evaluate the anti-chemotactic activity of BK1.3. Representative dose–response curves are shown for BK1.3 (green). The scrambled control peptide, BK1.3-SCR, is referred to as the sham throughout this study (grey). Data are presented as mean ± SD of three technical replicates. The IC_50_ of BK1.3 was calculated using a 4-parameter log-logistic model fitted to the inhibitor response curve. **B** An in vivo model was employed to assess the anti-chemotactic activity of BK1.3. Male C57BL/6 J mice were administered 2 mg/kg LPS intranasally to induce acute lung inflammation. Intraperitoneal treatments were given directly after LPS-administration and 9 h later, with mice receiving either the control peptide BK1.3-SCR (*n* = 12) or peptide BK1.3 (*n* = 12). Mice were euthanized after 24 h. The final analysis time point was reached by 11 mice in the BK1.3-SCR group and 12 mice in the BK1.3 group, respectively. 1 mouse in the BK1.3-SCR group prematurely met termination criteria and was not further analyzed. Bronchoalveolar lavage fluid (BALF) was collected and analyzed by flow cytometry. **C** The gating strategy used to identify leukocytes, Ly6G^+^ neutrophils, Ly6C^+^ monocytes, and CD3^+^ T cells is depicted. **D** Absolute cell counts of leukocytes, Ly6C^+^ monocytes, Ly6G^+^ neutrophils, and CD3^+^ T cells in BALF are presented for the two treatment groups. Data for total cells in BALF are shown as boxplots with individual values and whiskers indicating minimum to maximum, alongside the mean. Comparisons were made between BK1.3-SCR control peptide-treated mice and the BK1.3 study arm using a two-tailed unpaired *t*-test without corrections for multiple comparisons. Asterisks (*) indicate significant results with *p* < 0.05, ns indicates non-significant results

Subsequently, we synthesized the Evasin-derived peptide BK1.3 and a corresponding control peptide BK1.3 SCR according to the published amino acid sequence and the methods detailed in the materials and methods section. Prior to evaluating BK1.3 in our preclinical murine CVB3 infection model, we needed to ensure the quality and efficacy of the synthesized peptide batch. First, we assessed the ability of the BK1.3 batch to inhibit CCL8-driven chemotaxis in a THP-1 cell migration assay (Fig. 2A). The dose–response peptide titration curve revealed inhibition of CCL8-driven cell migration with increasing BK1.3 concentrations and a calculated IC_50_ value for BK1.3 of approximately 4.2 nM, consistent with previously reported values [23]. As expected, the control peptide (BK1.3 SCR) exhibited no activity in the assay.

To further confirm the efficacy of BK1.3, we employed an in vivo model of acute LPS-induced lung inflammation. C57BL/6 J mice were treated intranasally with LPS, followed by intraperitoneal administration of the BK1.3 batch, or the control peptide, mirroring the administration route intended for the CVB3 infection model (Fig. 2B). After 24 h, bronchoalveolar lavage fluid (BALF) was collected, and immune cell counts were quantified via flow cytometry. In comparison to the control group, treatment with BK1.3 led to a clear reduction in the total CD45^+^ leukocyte count in BALF, as well as a decrease in Ly6C^+^ monocytes and Ly6G^+^ neutrophils (Fig. 2C/D). These quality control experiments confirm that the synthesized BK1.3 batch effectively inhibits chemotaxis. Combined with the validation of the chemokine expression profile in the liver (Fig. 1D/E), this provides a solid foundation for testing the novel chemokine-binding peptide BK1.3 in the murine CVB3 infection model.

### Early systemic impairment and hepatitis remain unaffected under BK1.3 treatment

**Fig. 3:**
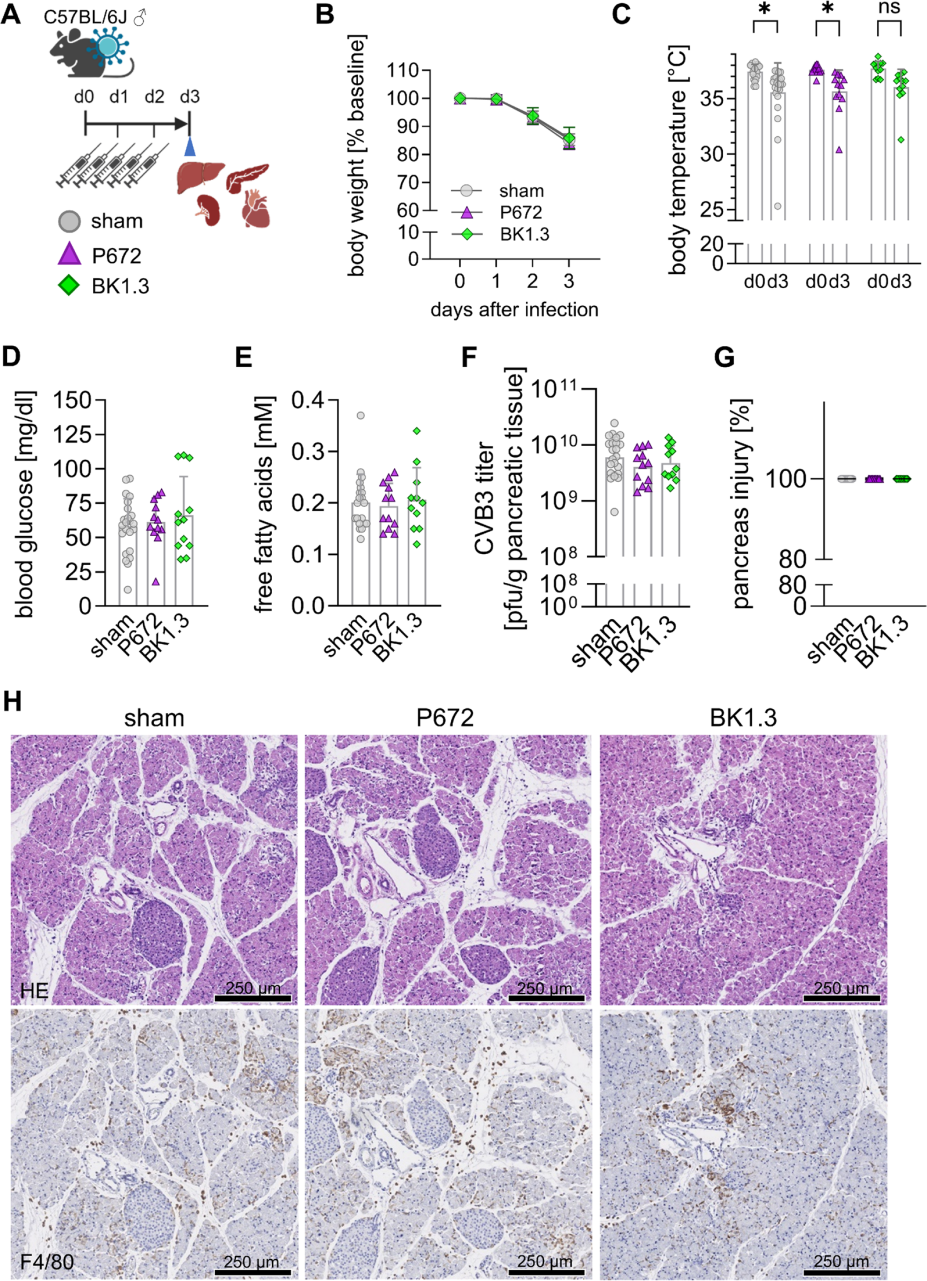
Early systemic compromise and pancreatitis remain unaffected under BK1.3 treatment. **A** Male C57BL/6 J mice were infected with CVB3. Treatments with sham (scrambled BK1.3-SCR control peptide, *n* = 23), P672 (*n* = 12), or BK1.3 (*n* = 12) began 12 h post-infection and were repeated every 12 h until euthanization on day 3 post-infection. All mice reached the final analysis time point in each group. No mice had to be excluded from the experiment due to prematurely met termination criteria. **B** Body weight was measured daily (sham, *n* = 23; P672, *n* = 12; BK1.3, *n* = 12). Data are presented as mean ± SD of relative body weight, normalized to baseline values on day 0. Statistical analysis was performed using one-way ANOVA, revealing no significant results. **C** Body temperature was recorded on day 0 and day 3 (sham, *n* = 23; P672, *n* = 12; BK1.3, *n* = 12). Statistical analysis was performed using two-way ANOVA with Sidak’s post hoc analysis. **D** Blood glucose levels were assessed on day 3 post-infection (sham, *n* = 23; P672, *n* = 12; BK1.3, *n* = 12). **E** Free fatty acid levels were measured 3 days post-infection (sham, *n* = 20; P672, *n* = 11; BK1.3, *n* = 12). **F** Viral load in pancreas tissue was quantified on day 3 post-infection using a Plaque Assay (sham, *n* = 23; P672, *n* = 12; BK1.3, *n* = 11). **G** Pancreas tissue sections were stained with hematoxylin and eosin (HE), and a pathologist scored pancreatic injury from 0 to 100% (sham, *n* = 23; P672, *n* = 12; BK1.3, *n* = 12). Data for **D–G** were analyzed using one-way ANOVA, revealing no significant results. **H** Representative micrographs of pancreas tissue sections stained with HE and immunohistochemistry for F4/80 are shown for sham-, P672-, and BK1.3-treated groups on day 3 post-infection. A black scale bar represents 250 µm

We initially explored the safety of BK1.3 treatment during the acute phase of murine CVB3 infection by administering it intraperitoneally to C57BL/6 J mice infected with CVB3, alongside a sham treatment with scrambled BK1.3-SCR control peptide and the Evasin protein P672 as a positive control (Fig. 3A). Given the broader binding spectrum of P672 and its higher chemokine affinities, it was used to detect potential adverse effects of chemokine network inhibition. We focused on day 3 post-infection markers of acute CVB3 infection. No significant differences were observed in body weight, onset of hypothermia, hypoglycemia, or free fatty acid levels across the treatment groups by day 3 post-infection (Fig. 3B-E), indicating that early systemic impairment was not exacerbated by the treatments. Furthermore, despite the potential for compromised virus control due to chemokine network inhibition, virus load in pancreatic tissue remained stable across all groups (Fig. 3F). Histological analysis confirmed severe but consistent injury levels in the pancreatic tissues across all experimental groups, regardless of treatment (Fig. 3G, H). Additionally, the presence of F4/80^+^ cells, which are mostly macrophages, in the pancreatic tissues was similarly elevated across all conditions, indicating no significant impact from the treatment interventions on this measure of immune infiltration. These findings suggest that BK1.3, like its parent protein P672, does not adversely affect early systemic responses or the progression of pancreatitis during acute CVB3 infection, supporting its potential safety and efficacy in targeting the chemokine network without enhancing viral pathogenicity.

**Fig. 4:**
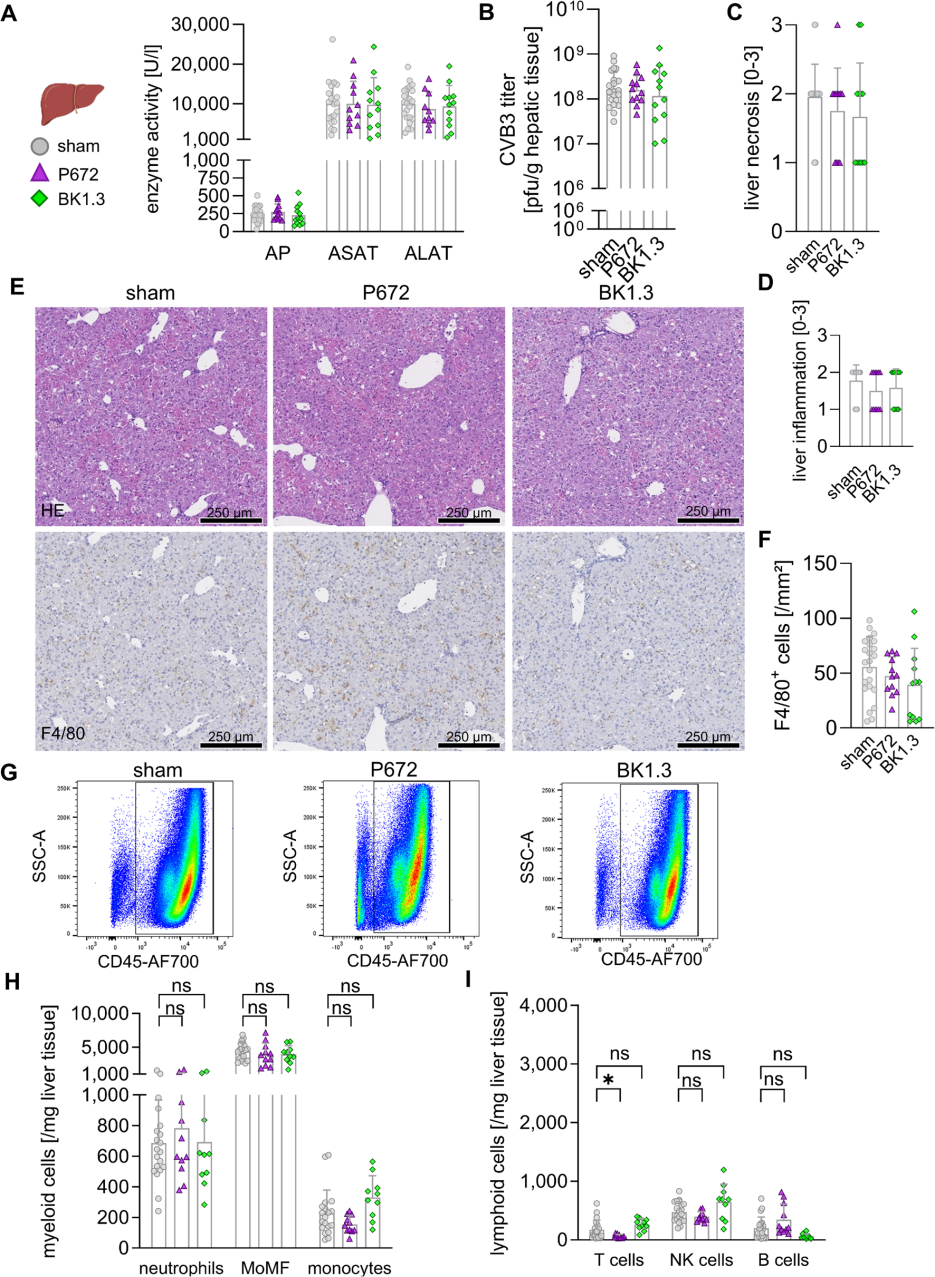
Regardless of P672 or BK1.3. treatment, CVB3 causes severe hepatic damage with predominantly myeloid immune cell infiltration. Male C57BL/6 J mice were infected with CVB3 and treated with sham (scrambled BK1.3-SCR control peptide, *n* = 23), P672 (*n* = 12) or BK1.3 (*n* = 12) starting 12 h post-infection until euthanization on day 3 post-infection. All mice reached the final analysis time point in each group. No mice had to be withdrawn from the experiment due to prematurely met termination criteria. **A** Serum enzyme activities of alkaline phosphatase (AP), aspartate-aminotransferase (ASAT) and alanine-aminotransferase (ALAT) were determined on day 3 post infection (sham, *n* = 23; P672, *n* = 12; BK1.3, *n* = 12). **B** Viral load in liver tissue was quantified on day 3 post-infection using a Plaque Assay (sham, *n* = 22; P672, *n* = 12; BK1.3, *n* = 12). **C** Semiquantitative scoring (0–3) of liver necrosis in hematoxylin and eosin-stained liver tissue sections (day 3) was performed by a pathologist (sham, *n* = 23; P672, *n* = 12; BK1.3, *n* = 12). **D** Similarly, semiquantitative scoring (0–3) of liver inflammation was carried out. **E** Representative micrographs of liver tissue section stained with HE and immunohistochemistry against F4/80 are shown for sham-, P672- and BK1.3-treated mice at day 3 post-infection. 250 µm length is indicated with a black scale bar. **F** Quantification of F4/80^+^ nucleated cells per mm^2^ in immunohistochemistry staining (sham, *n* = 23; P672, *n* = 12; BK1.3, *n* = 12). **G** Immune cell infiltration into the liver tissue on day 3 post-infection was investigated by means of multicolor flow cytometry. Representative images of the corresponding leucocyte gates are depicted for each treatment group. Average leukocyte counts per milligram liver tissue were 7421, 6694 and 7273 for the sham-, P672- and BK1.3-treated mice, respectively. **H** Quantification of the following myeloid subpopulations per milligram liver tissue: neutrophils, monocyte-derived macrophages (MoMF) and monocytes. **I** Quantification of the following lymphoid subpopulations per milligram liver tissue: T cells, natural killer (NK) cells and B cells. Data for (A, B, C, D, F, H, and I) were analyzed using one-way ANOVA (unpaired, unmatched), followed by Dunnett’s multiple comparison test when indicated. The sham group was compared to either P672- or BK1.3-treated animals. Significant results (*p* < 0.05) are marked with an asterisk (*), while non-significant results are labeled as “ns”. Unless otherwise stated, results were not significant

To assess the impact of the BK1.3 peptide on liver pathology during acute CVB3 infection, we utilized the established experimental framework (Fig. 3A). By day 3 post-infection, we observed significant elevations in serum enzyme activities-alkaline phosphatase (AP), aspartate aminotransferase (ASAT), and alanine aminotransferase (ALAT)-indicating substantial hepatocyte necrosis (Fig. 4A). Importantly, viral loads in liver tissue remained consistent across all treatment groups (Fig. 4B). The administration of BK1.3 or the control peptide P672 did not exacerbate any of these hepatic injury markers. Histopathological examination corroborated these findings, revealing extensive hepatocellular necrosis consistent with the acute phase of CVB3 infection (Fig. 4E). Semiquantitative assessments of liver necrosis and inflammation showed no significant differences among the sham, BK1.3, and P672 treatment groups (Fig. 4C, D), further indicating that BK1.3 does not intensify hepatic pathology. Immunohistochemical analysis identified a similar abundance of F4/80 + macrophages across all treatment groups (Fig. 4E), with consistent F4/80^+^ cell density regardless of treatment with BK1.3 or P672 (Fig. 4F). To further elucidate the hepatic immune cell composition during acute CVB3 infection, we employed multicolor flow cytometry, which revealed no significant differences in the population of CD45^+^ leukocytes among the treatment groups (Fig. 4G). Detailed characterization of these immune cells indicated that the hepatic inflammatory response was predominantly driven by myeloid cells, particularly CD11b^high^ F4/80^intermediate^ monocyte-derived macrophages, with smaller contributions from Ly6G^+^ neutrophils and Ly6C^+^ monocytes (Fig. 4H). Lymphoid cells, including T cells, B cells, and NK cells, were also present but to a lesser extent (Fig. 4I). Notably, BK1.3 treatment did not alter the composition or magnitude of immune cell infiltration, suggesting that systemic chemokine inhibition does not affect the immune response in the liver during acute CVB3 infection. In conclusion, these results demonstrate that BK1.3 does not exacerbate liver injury or alter hepatic immune cell infiltration during the acute phase of CVB3 infection. This supports the potential safety of BK1.3 as a chemokine network-targeting therapeutic.

### Early cardiac dysfunction during acute CVB3 Infection is unaffected by BK1.3 treatment

**Fig. 5:**
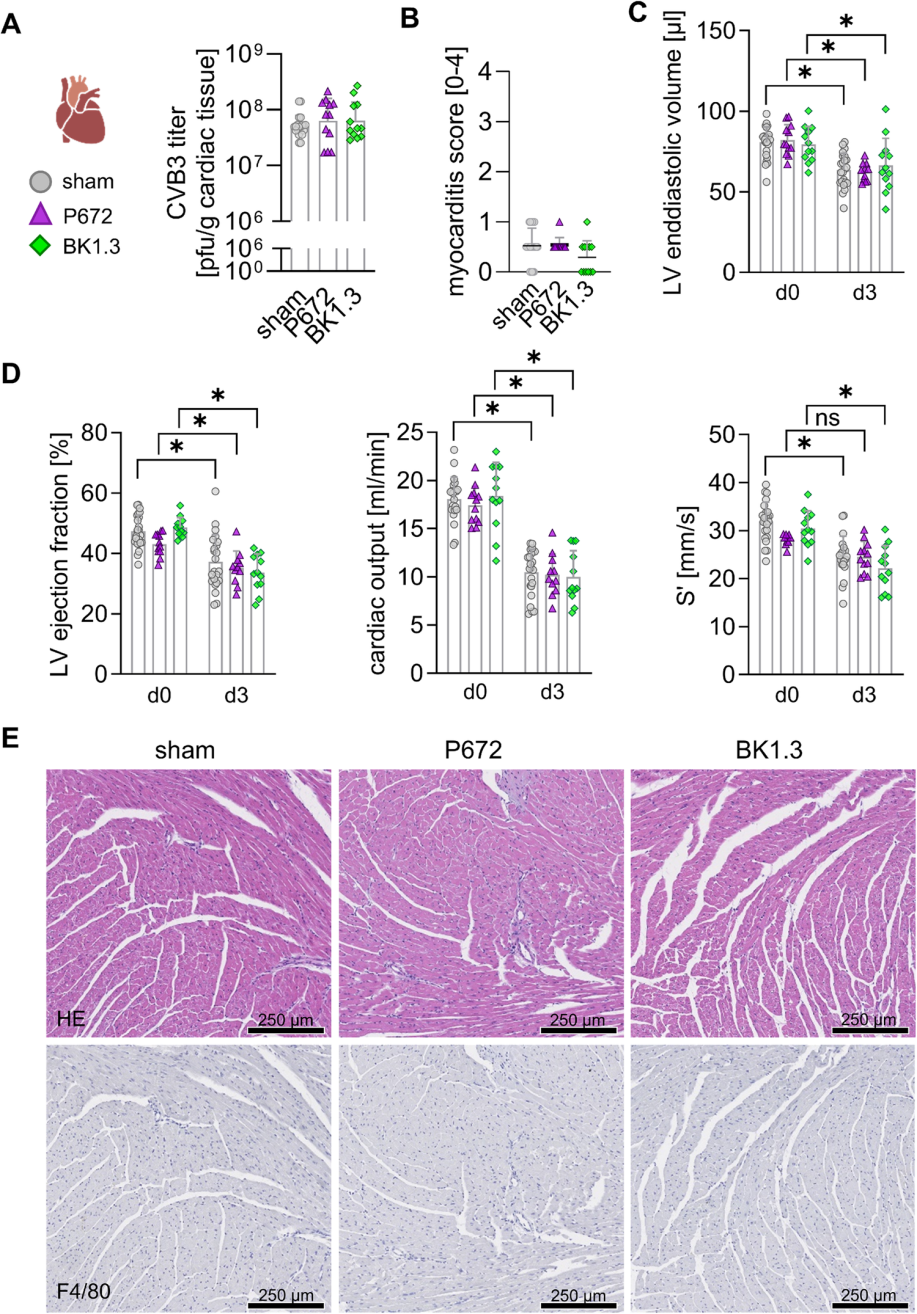
Cardiac function is compromised early during CVB3 infection, but BK1.3 treatment does not exacerbate this deterioration. Male C57BL/6 J mice were intraperitoneally infected with CVB3. Treatment with sham (scrambled BK1.3-SCR control peptide, *n* = 23), P672 (*n* = 12), or BK1.3 (*n* = 12) began 12 h post-infection and was administered every 12 h until euthanasia on day 3 post-infection. All mice reached the final analysis time point in each group. No mice had to be withdrawn from the experiment due to prematurely met termination criteria. Echocardiography was performed at baseline and on day 3 post-infection. **A** Viral load in heart tissue on day 3 post-infection was assessed via Plaque Assays (sham, *n* = 21; P672, *n* = 12; BK1.3, *n* = 12). **B** Histological samples were stained with hematoxylin and eosin. Myocarditis severity was evaluated semi-quantitatively by a pathologist (sham, *n* = 23; P672, *n* = 12; BK1.3, *n* = 12). Data for (A/B) were analyzed using one-way ANOVA, with no significant differences observed. **C**, **D** Left ventricular (LV) end-diastolic volume, ejection fraction, cardiac output, and systolic wall motion velocity (S’) were measured at baseline and on day 3 post-infection using echocardiography (sham, *n* = 23; P672, *n* = 11; BK1.3, *n* = 12). For these repeated measures, a paired two-way ANOVA followed by Sidak's multiple comparison test between baseline and day 3 values in each group was performed. **E** Representative micrographs of heart tissue sections stained with HE and immunohistochemistry against F4/80 are shown for the sham-, P672-, and BK1.3-treated groups on day 3 post-infection. A black scale bar indicates 250 µm

Significant cardiac inflammation typically develops later in CVB3 infection; however, viral cytotoxicity and chemokine release also emerge in the heart by day 3 post-infection. To assess the impact of the chemokine-binding peptides BK1.3 and P672 on cardiac function at this early phase, we conducted a comprehensive analysis, including echocardiography, on day 3 post-infection. Virus load measurements confirmed ongoing replication in heart tissue across all groups, with no significant differences between the sham, BK1.3, and P672 treatments (Fig. 5A). Histological analysis showed minimal cardiac inflammation, consistent with the early infection stage, and no significant inflammatory cell infiltration, particularly of F4/80^+^ macrophages, was observed across all groups (Fig. 5B, E). This absence of significant inflammation at day 3, despite clear cardiomyocyte damage, suggests that cardiac dysfunction at this phase is primarily driven by direct virus-induced effects and attributed to systemic vaso- and cardiosuppressive mediators rather than by cardiac inflammation. Echocardiographic assessments revealed a marked decline in cardiac function by day 3, characterized by reduced left ventricular (LV) end-diastolic volume, ejection fraction, and cardiac output, indicating impaired cardiac performance (Fig. 5C, D; Table 1). The reduction in LV filling volume is consistent with previously reported hypovolemic conditions in this model, contributing to the decreased cardiac output [34]. Additionally, S’ velocity, a parameterless dependent on preload conditions, was also significantly reduced, confirming the early onset of cardiac dysfunction (Fig. 5D). The administration of BK1.3 or P672 did not exacerbate cardiac dysfunction, suggesting that BK1.3 is safe with respect to early cardiac function, maintaining virus control without worsening cardiac pathology during the early stages of infection.

**Table 1:** Cardiac output impairment induced by acute CVB3 Infection

	Sham		BK1.3		P672	
	Baseline	Day 3	Baseline	Day 3	Baseline	Day 3
Heart rate [bpm]	486.1 ± 35.9	452.2 ± 44.1*	474.3 ± 36.4	453.4 ± 49.7	496.1 ± 41.9	465 ± 47.1
LVEDV [µl]	80.6 ± 9.6	63.5 ± 10.5*	79.5 ± 11.2	66.5 ± 16.8*	82.1 ± 9.5	62.3 ± 5.7*
LVESV [µl]	42.7 ± 7.7	42.2 ± 8.3	37.5 ± 10.6	44.5 ± 13.3*	46.8 ± 6.5	40.4 ± 5.4*
Stroke volume [µl]	37.3 ± 3.7	23 ± 4.4*	38.6 ± 5.9	22 ± 5.7*	35.3 ± 4.9	21.9 ± 3.9*
Cardiac output [ml/min]	18.1 ± 2.3	10.5 ± 2.4*	18.4 ± 3.5	10 ± 2.7*	17.4 ± 2.0	10.2 ± 2.2*
Trace LVEF [%]	47.4 ± 5.4	37.2 ± 9.1*	48.6 ± 3.3	33.7 ± 6.1*	43.1 ± 3.7	35.2 ± 5.6*
FS LV length [%]	14 ± 2.1	14.1 ± 2.6	12.7 ± 1.4	11.3 ± 2.6 ^#^	13.2 ± 0.8	14.4 ± 1.4
FAC [%]	50.1 ± 4.9	41.7 ± 7.4*	49.4 ± 3.7	37.2 ± 5.0*	50.2 ± 5.8	42.9 ± 5.4*
LVAWd [mm]	0.8 ± 0.09	0.9 ± 0.15*	0.7 ± 0.11	0.7 ± 0.14^#^	0.8 ± 0.13	0.9 ± 0.11*
LVPWd [mm]	0.6 ± 0.08	0.7 ± 0.12*	0.6 ± 0.08	0.6 ± 0.11^#^	0.6 ± 0.10	0.7 ± 0.11
LVIDd [mm]	4.3 ± 0.23	3.7 ± 0.38*	4.3 ± 0.12	4.1 ± 0.35^#^	4.3 ± 0.37	3.5 ± 0.21*
LV Mass [mg/g]	4 ± 0.39	4.2 ± 0.66	3.7 ± 0.59	3.6 ± 0.63^#^	4.2 ± 0.61	4.1 ± 0.54
MV E [mm/s]	832 ± 109.4	477.6 ± 72.3*	886 ± 75.4	467.6 ± 91.9*	804.6 ± 106.0	513.9 ± 57.7*
MV A [mm/s]	543.5 ± 93.8	405.9 ± 63.9*	580.2 ± 75.9	444.3 ± 50.2*	505.1 ± 88.9	380.4 ± 40.6*
MV E/A	1.6 ± 0.20	1.2 ± 0.22*	1.6 ± 0.29	1.1 ± 0.26*	1.6 ± 0.17	1.3 ± 0.14*
IVRT [ms]	15.3 ± 3.2	18.5 ± 4.4*	15 ± 2.7	22.1 ± 3.7*^#^	17.7 ± 2.5	16.2 ± 3.3
IVCT [ms]	13.8 ± 3.3	15.5 ± 3.3	12.8 ± 3.6	16.7 ± 2.4*	15 ± 2.2	15.8 ± 2.4
AET [ms]	45.7 ± 3.9	40.2 ± 6.5*	48.4 ± 4.1	43.5 ± 6.0*	45.6 ± 4.4	39 ± 5.6*
MV E Decel Time [ms]	20.3 ± 5.9	21.3 ± 4.0	20.6 ± 6.5	21.7 ± 2.9	22.3 ± 6.1	19.6 ± 2.2
Tei-Index	0.6 ± 0.13	0.9 ± 0.15*	0.6 ± 0.11	0.9 ± 0.10*	0.7 ± 0.07	0.8 ± 0.14*
MV E' [mm/s]	25.1 ± 5.9	10.5 ± 3.3*	26.2 ± 4.8	11.1 ± 2.8*	22.9 ± 4.0	12 ± 1.8*
MV A' [mm/s]	29.1 ± 3.4	18.5 ± 4.9*	31.7 ± 5.8	21 ± 4.7*	27 ± 4.2	17.5 ± 3.0*
MV E'/A'	0.8 ± 0.13	0.6 ± 0.19*	0.8 ± 0.10	0.6 ± 0.18*	0.9 ± 0.19	0.7 ± 0.14*
MV E/E'	34.5 ± 7.3	49.1 ± 12.6*	34.9 ± 8.0	44.4 ± 12.6*	35.8 ± 5.8	43.4 ± 6.8*
S' [mm/s]	32.0 ± 4.0	24.6 ± 4.3*	30.4 ± 3.7	22.2 ± 4.7*	28.2 ± 1.2	24.6 ± 3.2*

C57BL/6 J mice were infected with 10^5^ PFU CVB3 and treated with either sham (scrambled BK1.3-SCR control peptide), (*n* = 23), BK1.3 (*n* = 12), or P672 (*n* = 12). Echocardiographic assessments were performed at baseline (2 days prior to infection) and on day 3 post-infection. Echocardiographic parameters are presented as mean ± SD. Statistical analyses were conducted using a repeated measures mixed-effects model, followed by Tukey’s test for multiple comparisons. Statistically significant results (*p* < 0.05) are indicated by * for comparisons between baseline and day 3 measurements within each treatment group, and by # for comparisons between the sham-treated group and either the BK1.3 or P672-treated groups on day 3

LVEDV: Left Ventricular End-Diastolic Volume; LVESV: Left Ventricular End-Systolic Volume; LVEF: Left Ventricular Ejection Fraction; FS LV: Fractional Shortening of the Left Ventricle; FAC: Fractional Area Change; LVAWd: Left Ventricular Anterior Wall in Diastole; LVPWd: Left Ventricular Posterior Wall in Diastole; LVIDd: Left Ventricular Internal Diameter in Diastole; LV Mass: Left Ventricular Mass; MV: Mitral Valve; IVRT: Isovolumetric Relaxation Time; IVCT: Isovolumetric Contraction Time; AET: Aortic Ejection Time; MV E Decel: Mitral Valve E Deceleration; MV E Decel Time: Mitral Valve E Deceleration Time

### Immune cell mobilization and activation in the spleen during acute CVB3 infection under chemokine inhibition

**Fig. 6:**
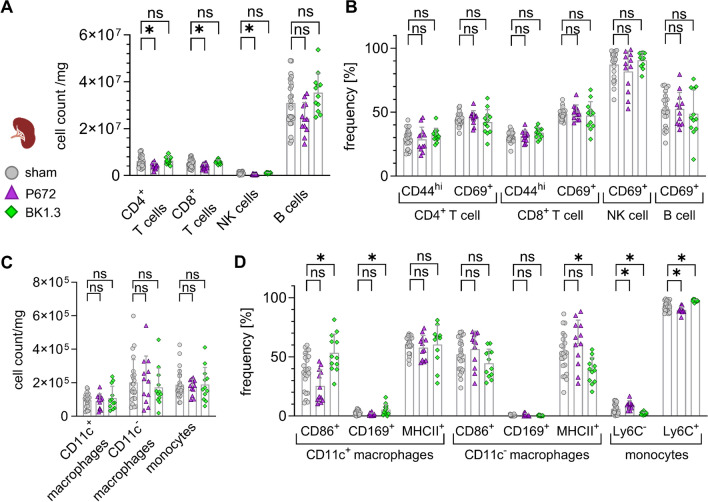
BK1.3 treatment does not affect immune cell abundance and activation in the spleen during CVB3 infection. C57BL/6 J mice were intraperitoneally infected with CVB3 and treated as described above. **A** Absolute immune cell counts per mg of splenic tissue were measured for the following lymphoid subpopulations: CD4^+^ T cells, CD8^+^ T cells, natural killer (NK) cells, and B cells (sham, *n* = 23; P672, *n* = 12; BK1.3, *n* = 12). **B** Immune cell activation was assessed in these lymphoid subpopulations by evaluating the expression of activation markers CD44 and CD69. Data are presented as the proportion of activation marker-positive cells relative to the corresponding immune cell subpopulation. **C** Absolute immune cell counts per mg of splenic tissue were measured for the following myeloid subpopulations: CD11c^+^ macrophages, CD11c^−^ macrophages, and monocytes (sham, *n* = 23; P672, *n* = 12; BK1.3, *n* = 12). **D** Immune cell activation was assessed in these myeloid subpopulations by evaluating the expression of activation markers CD86, CD169, MHCII, and Ly6C. Data are presented as the proportion of activation marker-positive cells relative to the corresponding immune cell subpopulation. Data for (A-D) were analyzed using one-way ANOVA (unpaired, unmatched), followed by Dunnett’s multiple comparison test. The sham group was compared to either P672- or BK1.3-treated animals. Significant results (*p* < 0.05) are indicated by an asterisk (*), while non-significant results are marked as “ns.”

During acute CVB3 infection, chemokines are crucial for the mobilization and activation of immune cells in lymphoid organs such as the spleen. Thus, assessing splenic immune cell dynamics is vital when evaluating the impact of the chemokine-binding compound BK1.3. Our analysis demonstrated that immune cell mobilization was maintained under chemokine inhibition with BK1.3 or P672. Lymphoid immune cell counts, including CD4^+^ T cells, CD8^+^ T cells, NK cells, and B cells, were consistent across all experimental groups (Fig. 6A). Similarly, myeloid subpopulations, such as CD11c^+^ macrophages, CD11c^−^ macrophages, and monocytes, showed no significant differences between groups (Fig. 6C). Additionally, the activation levels of both lymphoid and myeloid subpopulations remained unaffected by treatment with either BK1.3 or P672 (Fig. 6B, D).

### Evaluation of BK1.3's effects on organ health during subacute CVB3 infection

**Fig. 7:**
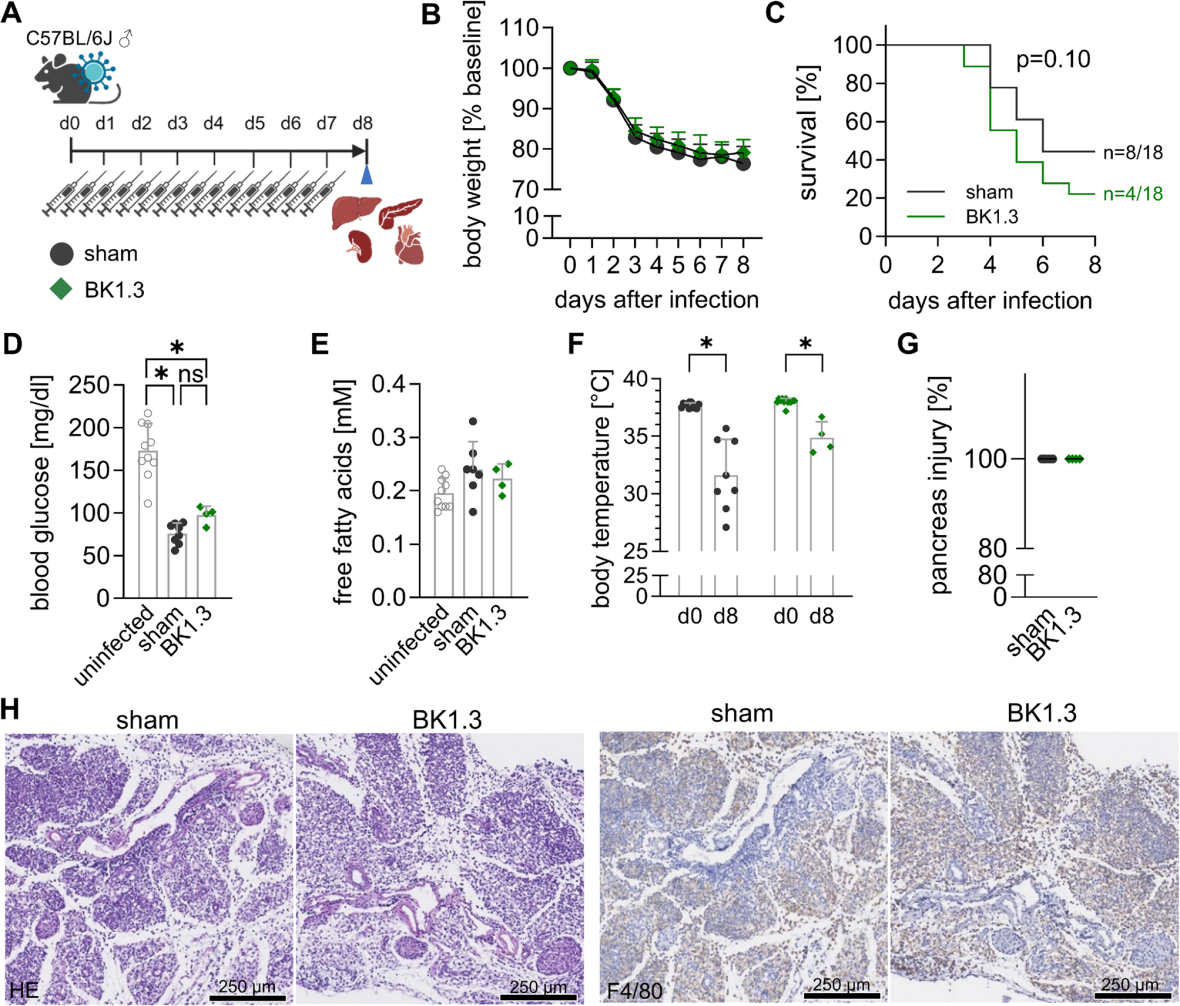
Prolonged BK1.3 treatment during the subacute phase of CVB3 infection. **A** Male C57BL/6 J mice were intraperitoneally infected with CVB3. Treatment with sham (scrambled BK1.3-SCR control peptide, *n* = 18), or BK1.3 (*n* = 18) began 12 h post-infection and was administered every 12 h until euthanasia on day 8 post-infection. This final analysis time point was reached by 8 mice in the sham group and 4 mice in the BK1.3 group, respectively. 10 mice in the sham and 14 mice in the BK1.3 group prematurely met termination criteria and were not further analyzed. **B** Body weight of the mice was measured daily (sham, *n* = 18; BK1.3, *n* = 18). Data are shown as mean ± SD relative body weight, normalized to body weight measured at baseline on day 0. Data were analyzed using paired 2-way ANOVA, revealing no differences between the treatment groups. **C** Survival of the mice from baseline until day 8 post-infection was assessed using the Log-rank (Mantel-Cox) test, which yielded a p-value of 0.10 (sham, *n* = 18; BK1.3, *n* = 18). **D** Blood glucose levels were determined on day 8 post-infection (sham, *n* = 8; BK1.3, *n* = 4), with data from uninfected animals (*n* = 10) also presented. **E** Free fatty acid levels were measured on day 8 post-infection (sham, *n* = 7; BK1.3, *n* = 4), alongside data from uninfected animals (*n* = 10). Data in (D) and (E) were analyzed using one-way ANOVA, followed by Tukey multiple comparisons of all groups. Significant results with *p* < 0.05 are indicated by asterisk (*), non-significant results with ns. One-way ANOVA in (E) yielded no significant results. (**F**) Body temperature was recorded at baseline and on day 8 post-infection, and these data were analyzed using paired 2-way ANOVA followed by Tukey’s post hoc test (sham, *n* = 18; BK1.3, *n* = 18). **G** Pancreas tissue sections stained with HE, were scored by a pathologist for pancreas injury (0 to 100%) on day 8 post-infection. The scoring data were also analyzed using an unpaired *t*-test (sham, *n* = 8; BK1.3, *n* = 4). **H** Representative micrographs of pancreas tissue sections stained with HE and immunohistochemistry against F4/80 are shown for the sham- and BK1.3-treated groups on day 8 post-infection. A black scale bar indicates a length of 250 µm

Our results confirm that BK1.3 effectively inhibits key chemokines without exacerbating systemic impairment, organ damage, or altering immune responses during acute CVB3 infection. With its safety profile established, we proceeded to assess the therapeutic potential of BK1.3 for viral myocarditis, focusing on its ability to mitigate myocardial inflammation and damage. To this end, we tested BK1.3 during the subacute phase of CVB3 infection. Male C57BL/6 J mice were infected with CVB3 and received intraperitoneal injections of either sham (scrambled BK1.3-SCR control peptide) or BK1.3 every 12 h until analysis on day 7 for echocardiography and euthanization on day 8, when peak myocardial inflammation was expected (Fig. 7A). The decrease in body weight observed on day 3 post-infection persisted until day 8, with no significant differences in the BK1.3-treated group (Fig. 7B). As the CVB3 infection progressed beyond day 3, some animals showed worsening symptoms. The overall survival rate did not differ significantly between the BK1.3-treated and sham-treated groups (Fig. 7C). Perturbations in plasma nutrients persisted on day 8 post-infection, with more pronounced hypoglycemia and higher free fatty acid levels in the sham-treated group compared to the BK1.3-treated group, although these differences were not statistically significant (Fig. 7D, E). Hypothermia, similar to weight loss, continued to progress until day 8 post-infection (Fig. 7F).

We then examined organs severely affected during the acute stage of CVB3 infection, focusing on the subacute phase. First, we examined the pancreatic tissue. Ongoing pancreatic destruction was observed in both groups and F4/80^+^ cells were dispersed throughout the pancreatic tissue (Fig. 7G, H), indicating immune cell infiltration likely involved in clearing debris and initiating tissue restoration.

**Fig. 8:**
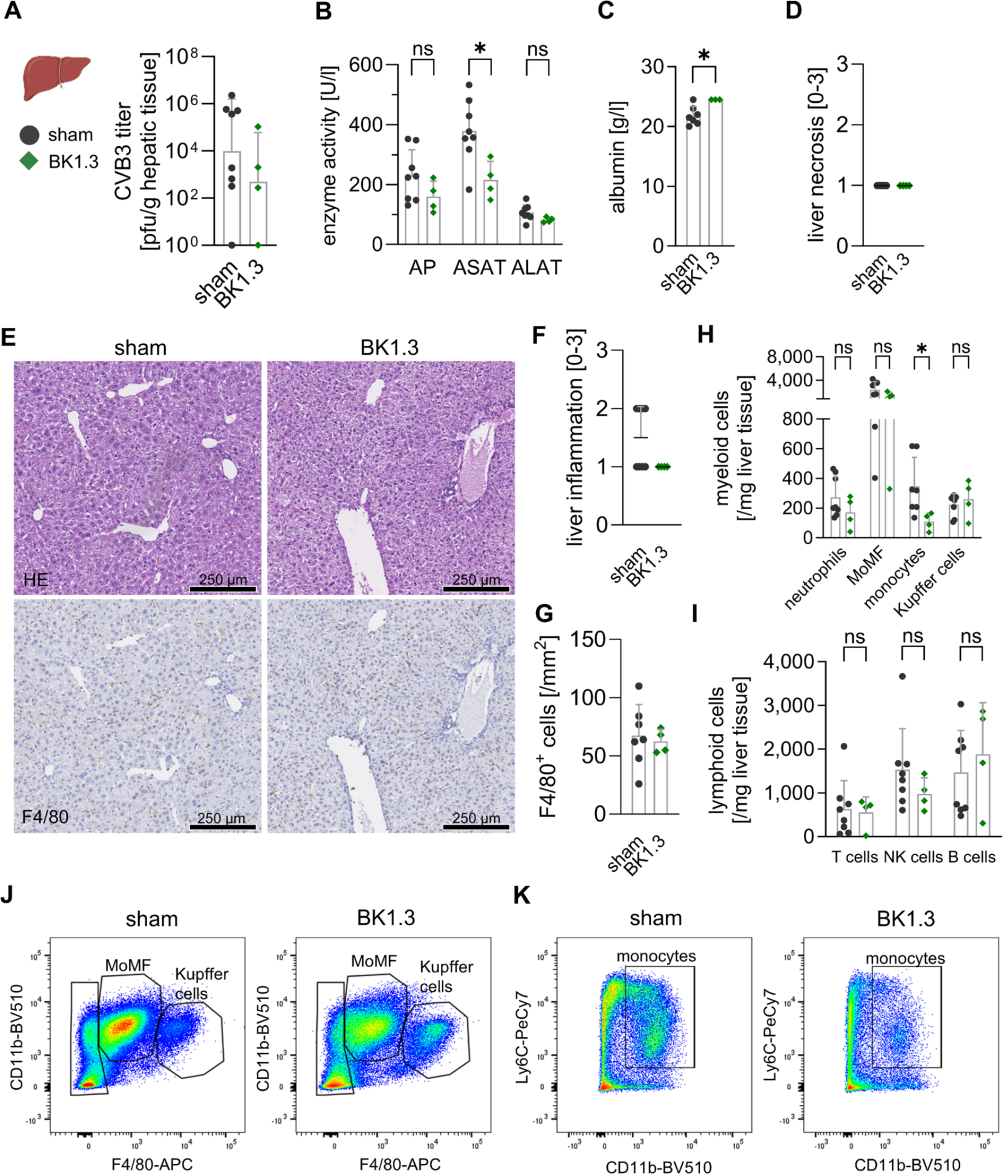
BK1.3 treatment facilitates restoration of liver function with anti-inflammatory effects. Male C57BL/6 J mice were infected with CVB3 and treated with sham (scrambled BK1.3-SCR control peptide, *n* = 18), or BK1.3 (*n* = 18) until euthanization on day 8 post-infection. This final analysis time point was reached by 8 mice in the sham group and 4 mice in the BK1.3 group, respectively. 10 mice in the sham and 14 mice in the BK1.3 group prematurely met termination criteria and were not further analyzed. **A** Viral load in liver tissue on day 8 post-infection was investigated using a Plaque Assay (sham, *n* = 8; BK1.3, *n* = 4). **B** Serum enzyme activities of alkaline phosphatase (AP), aspartate-aminotransferase (ASAT), and alanine-aminotransferase (ALAT) were determined on day 8 post-infection (sham, *n* = 8; BK1.3, *n* = 4). **C** Serum albumin levels were measured on day 8 post-infection (sham, *n* = 8; BK1.3, *n* = 4). **D** Liver tissue sections were obtained from animals sacrificed on day 8 post-infection, stained with HE, and semi-quantitatively scored (0–3) for liver necrosis (sham, *n* = 8; BK1.3, *n* = 4). **E** Representative micrographs of liver tissue sections stained with HE and immunohistochemistry against F4/80 are shown for the sham- and BK1.3-treated groups on day 8 post-infection. A black scale bar indicates a length of 250 µm. **F** Liver inflammation was semi-quantitatively scored (0–3). **G** Immunohistochemical staining against F4/80 was quantified as F4/80^+^ nucleated cells per mm^2^ (sham, *n* = 7; BK1.3, *n* = 4). **H** Immune cell infiltration into the liver tissue on day 8 post-infection was analyzed using multicolor flow cytometry. Absolute immune cell counts per mg of liver tissue were quantified for the following myeloid subpopulations: neutrophils, monocyte-derived macrophages (MoMF), monocytes, and Kupffer cells. **I** Absolute immune cell counts per mg of liver tissue were similarly quantified for the following lymphoid subpopulations: T cells, natural killer (NK) cells, and B cells. **J** Representative images of MoMF and Kupffer cell gates are shown for the sham- and BK1.3-treated groups. The average MoMF count per mg of liver tissue was 2,357 in the sham group and 1,240 in the BK1.3-treated group. The average Kupffer cell count per mg of liver tissue was 224 in the sham group and 261 in the BK1.3-treated group. **K** Representative images of monocyte gating are shown for the sham- and BK1.3-treated groups. The average monocyte count per mg of liver tissue was 347 in the sham group and 109 in the BK1.3-treated group. Statistical analyses for panels A-D and F-I were conducted using an unpaired *t*-test. Significant results (*p* < 0.05) are marked with an asterisk (*), while non-significant results are labeled as “ns”. Unless otherwise indicated, the results were not significant

To assess liver restoration during the subacute phase of CVB3 infection under the influence of BK1.3, we analyzed hepatic tissue on day 8 post-infection using the established experimental framework as aforementioned. Virus clearance was unaffected by BK1.3 treatment, as indicated by similar hepatic virus loads between the BK1.3 and sham-treated groups (Fig. 8A). Importantly, serum markers of liver necrosis, which were drastically elevated during acute injury on day 3, showed significant reduction by day 8, with a trend towards faster normalization in BK1.3-treated animals. ASAT levels were notably lower in the BK1.3 group, while albumin levels were elevated, suggesting enhanced hepatic protein synthesis capacity (Fig. 8B, C). Histological analysis confirmed intact healing in BK1.3-treated mice, with no significant differences observed between the groups in terms of liver necrosis or inflammation (Fig. 8D-F). Immunohistochemical staining revealed comparable levels of F4/80^+^ cells across all groups, indicating consistent macrophage presence (Fig. 8G). Flow cytometry results complemented these findings, showing that immune cell infiltration was not adversely affected by BK1.3 treatment, with counts of monocyte-derived macrophages (MoMF), neutrophils, monocytes, and NK cells similar and reduced to those in the sham group (Fig. 8H-K). This consistency between immunohistochemical and flow cytometry results reinforces that BK1.3 does not impede the immune cell dynamics essential for liver recovery. Instead, the data suggest that BK1.3 supports a balanced immune response with intact infiltration of T and B cells during the subacute phase of CVB3 infection, maintaining effective liver restoration processes without negative impact.

### Impact of Evasin treatment on adaptation of liver metabolism during CVB3 infection

**Fig. 9:**
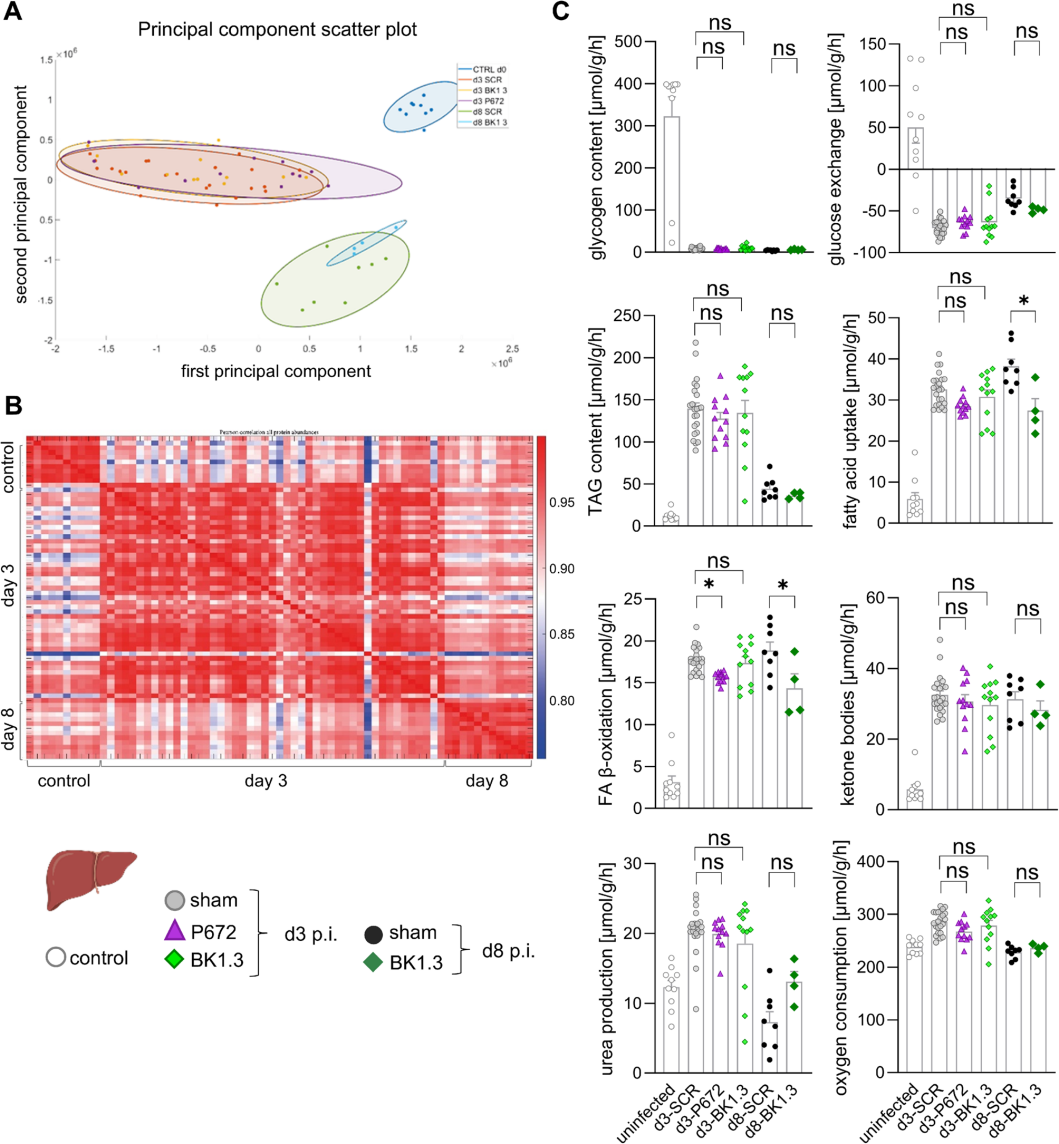
Impact of Evasin treatment on liver metabolism during CVB3 infection. **A** Principal component analysis (PCA) of the liver proteome from uninfected mice (control) and CVB3-infected mice treated with either sham (scrambled BK1.3-SCR control peptide), the Evasin protein P672 or the peptide BK1.3, and euthanized on days 3 and 8 post-infection. **B** Pearson correlation analysis matrix of the liver proteome across the different experimental groups. The matrix illustrates the degree of similarity between the proteomic profiles. Hierarchical cluster analysis of all proteins and all samples normalized so that for each protein the mean is 0 and the standard deviation is 1. Red and blue colors indicate increased and decreased abundance of the respective protein in each sample compared to the overall mean. There is a perfect separation between the livers of control and infected (early vs. subacute) animals. **C** Hepatokin1 modeling of liver metabolic processes based on individual blood glucose and fatty acid levels, illustrating the impact of CVB3 infection and Evasin treatments on hepatic metabolism. Metabolic functions are depicted in individual metabolic state. The data in panel C were analyzed by running a two-way ANOVA with time and treatment as factors. A statistically significant effect of time was observed across all panels. Where a significant effect of treatment was detected, post hoc testing was conducted to determine differences between groups. Significant results (*p* < 0.05) are marked with an asterisk (*), while non-significant results are denoted as “ns”. Group sizes were as follows: control, *n* = 10; d3 sham, *n* = 22; d3 P672, *n* = 12; d3 BK1.3, *n* = 12; d8 sham, *n* = 8; d8 BK1.3, *n* = 4

CVB3 infection significantly increases energy demands and alters plasma nutrient levels, necessitating adaptive changes in liver metabolism to maintain fuel supply. The liver’s role in supporting metabolic homeostasis during these conditions is well-established, with studies linking immune responses to metabolic adjustments in CVB3 infection [15, 16, 33]. This study aimed to assess whether Evasin treatments might disrupt liver metabolic adaptation during CVB3 infection. We conducted a detailed proteomic analysis of liver tissues using mass spectrometry. Principal component analysis (PCA) of the liver proteome revealed distinct clustering based on infection status: non-infected controls were clearly separated from infected groups (day 3 and day 8), indicating significant proteomic changes due to CVB3 infection. However, no significant clustering was observed based on treatment with P672 or BK1.3, suggesting these treatments did not induce additional proteomic alterations beyond those caused by the infection (Fig. 9A). The Pearson correlation analysis plot illustrates the degree of similarity between the proteomic profiles of the various conditions, with a high correlation indicating similar proteomic landscapes. The plot further supports the PCA findings, showing strong correlations between proteomes of infected groups treated with P672 or BK1.3 and those of untreated infected controls. This suggests that Evasin treatments do not significantly alter the overall proteomic profile of the liver during CVB3 infection, thereby supporting the notion that these treatments do not disrupt liver metabolic processes (Fig. 9B).

While the PCA and correlation matrix plot highlighted infection-related changes in the liver proteome, they did not reveal specific metabolic pathways. To assess whether BK1.3 treatments affect liver metabolism, we used the HEPATOKIN1 model [6], integrating proteomic data with glucose and free fatty acid plasma concentrations and calculated metabolic function in the liver (Fig. 9C). The model showed consistently low liver glycogen content at both day 3 and day 8 of infection, reflecting hypoglycemia. This was compensated by an elevated gluconeogenesis rate, particularly on day 3, which helped maintain fuel supply during the infection, consistent with increased urea production by the liver. By day 8, glucose production demand slightly decreased, indicating partial metabolic recovery. Additionally, we observed increased fatty acid uptake and storage, with elevated triglyceride (TAG) content on day 3, reflecting a shift toward elevated β-oxidation and increased ketone body production. Importantly, neither P672 nor BK1.3 treatments disrupted these metabolic adaptations, suggesting that the liver’s ability to meet the organism’s fuel demands remained intact throughout both the acute and recovery phases of infection. These findings support the safety of Evasin treatments, as they preserve essential metabolic functions during CVB3 infection.

### Targeted chemokine inhibition by BK1.3 mitigates myocardial inflammation and preserves cardiac function during subacute CVB3 infection

To investigate the effects of chemokine inhibition with BK1.3 on the myocarditis phenotype during the subacute phase of CVB3 infection, C57BL/6 J mice were treated twice daily with BK1.3 and analyzed on day 8 post-infection. Echocardiography was conducted both prior to infection and on day 7 post-infection to comprehensively assess cardiac function. It is important to note that the number of animals available for analysis at this time point was limited due to the high lethality of the infection model (Fig. 7C), particularly around day 4 and beyond. This limitation should be considered when interpreting the findings.

**Fig. 10:**
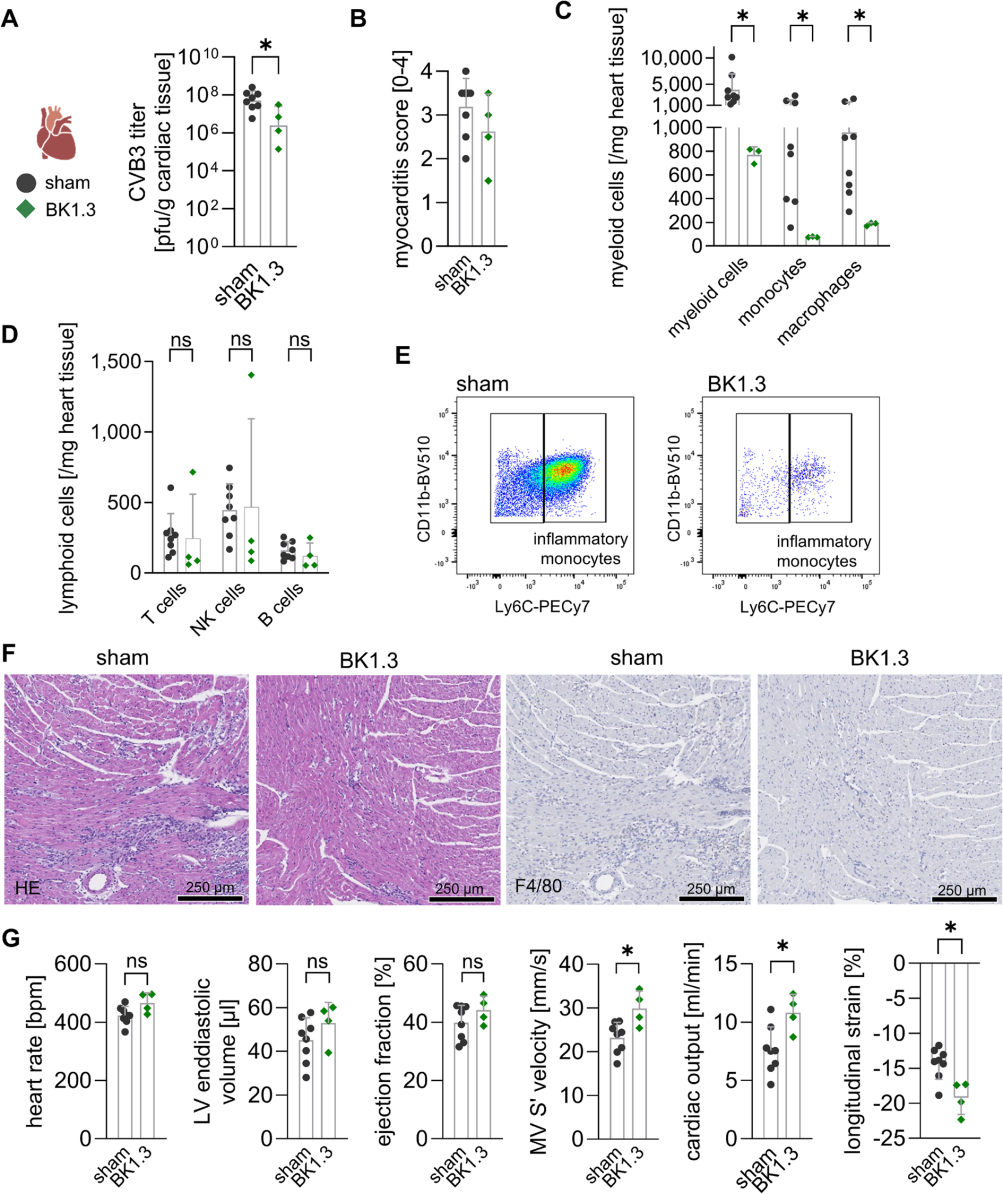
BK1.3 treatment reduces cardiac myeloid immune cell infiltration and helps preserve heart function. Male C57BL/6 J mice were infected with CVB3 intraperitoneally and treatment with sham (scrambled BK1.3-SCR control peptide, *n* = 18), or BK1.3 (*n* = 18) began 12 h post-infection and was repeated every 12 h until euthanization on day 8 post-infection. The final analysis time point was reached by 8 mice in the sham group and 4 mice in the BK1.3 group, respectively. 10 mice in the sham and 14 mice in the BK1.3 group prematurely met termination criteria and were not further analyzed. **A** Virus load in heart tissue on day 8 post-infection was analyzed using Plaque Assays (sham, *n* = 8; BK1.3, *n* = 4). **B** Semiquantitative myocarditis score ranging from 0 to 4 was assigned to cardiac HE-stained tissue sections (sham, *n* = 8; BK1.3, *n* = 4). **C** Immune cell infiltration into heart tissue on day 8 post-infection was investigated using multicolor flow cytometry. Absolute immune cell counts per mg of heart tissue were quantified for total myeloid cells, monocytes, and macrophages (sham, *n* = 8; BK1.3, *n* = 3). **D** Similarly, absolute immune cell counts per mg of heart tissue were quantified for the following lymphoid subpopulations: T cells, natural killer (NK) cells, and B cells (sham, *n* = 8; BK1.3, *n* = 4). **E** Representative images of inflammatory monocyte gates are shown for the sham- and BK1.3-treated groups. The average inflammatory monocyte count per mg of heart tissue was 1,086 in the sham group and 77 in the BK1.3-treated group. **F** Representative micrographs of heart tissue sections stained with HE and immunohistochemistry against F4/80 are shown for the sham- and BK1.3-treated groups on day 8 post-infection. A black scale bar indicates a length of 250 µm. **G** Echocardiography was performed at baseline and on day 7 post-infection. Heart rate left ventricular (LV) end-diastolic volume, ejection fraction, mitral valve (MV) S', cardiac output, and global longitudinal strain were measured on day 7 post-infection by echocardiography (sham, *n* = 8; BK1.3, *n* = 4). All statistical analyses for panels A-D and G were performed using an unpaired *t*-test

BK1.3 treatment resulted in a significant reduction of the virus load in heart tissue (Fig. 10A), somewhat inconsistent with the effects observed at the acute phase. Histological analysis revealed severe cardiac inflammation with substantial immune cell infiltration on day 8 post-infection (Fig. 10B, F). BK1.3-treated animals exhibited a slightly lower myocarditis score, but this reduction did not reach statistical significance. However, it is crucial to acknowledge the limitations of histology in this context. Myocarditis often presents with focal lesions that may not be consistently visible in all histological sections, potentially underestimating the extent of inflammation [20, 31]. In contrast, flow cytometry offers a more comprehensive assessment by quantifying immune cells across nearly the entire ventricle, providing a more accurate representation of immune cell infiltration. Detailed flow cytometry analysis revealed that the extensive recruitment of myeloid-derived immune cells observed in sham-treated animals was markedly reduced in BK1.3-treated mice (Fig. 10C-E). Notably, the infiltration of myeloid immune cells, including monocytes and macrophages, was significantly diminished. This is particularly relevant given that BK1.3 is designed to target chemokines such as CCL8, CCL7, CCL2, and CCL3, which are known to primarily affect myeloid cell trafficking [56]. Interestingly, although initial viral titers were similar across groups (Fig. 5A) and likely triggered both myeloid and lymphoid immune cell infiltration, the effects of BK1.3 treatment were specifically achieved by blocking the chemokines responsible for myeloid cell recruitment. This selective inhibition led to a significant reduction in the infiltration of these pro-inflammatory myeloid cells, while the infiltration of lymphoid cells, such as T cells, remained unaffected. The preservation of T cell infiltration, despite the reduced viral load, supports the hypothesis that unchecked myeloid cell infiltration may contribute to a pro-viral environment.

While the observed reduction in myeloid cell infiltration is indicative of BK1.3’s anti-inflammatory effects, it is essential to determine whether these cellular changes translate into functional benefits for the heart. To evaluate the clinical relevance of these anti-inflammatory effects, we conducted comprehensive echocardiographic assessments. Despite the reduction in myeloid cell infiltration, we needed to verify if this corresponded with improved cardiac performance. Echocardiography revealed persistent deterioration in left ventricular (LV) filling and reduced pump function in sham-treated mice, characterized by a low ejection fraction (EF), low cardiac output, and reduced S’ velocity, a key tissue Doppler parameter for assessing LV contractile function. In particular, global longitudinal strain (GLS), a highly sensitive tool for assessing LV function, especially in myocarditis with focal distribution patterns [20, 31], was significantly reduced in sham-treated infected mice compared to baseline, indicating impaired systolic function. In contrast, BK1.3-treated mice did not exhibit this reduction, with significant differences in GLS, S' velocity, and cardiac output between sham and BK1.3 groups on day 7 (Fig. 10G, Table 2). These findings confirm that the anti-inflammatory effects of BK1.3, particularly the reduction in myeloid cell infiltration, are functionally significant and lead to the preservation of cardiac function during the subacute phase of CVB3 infection. The use of flow cytometry, in particular, provided a more reliable and comprehensive quantification of immune cell dynamics than histology, thereby enhancing the robustness of these conclusions.

**Table 2:** Cardiac output impairment caused by myocardits during subacute CVB3 infection

	Sham		BK1.3	
	Baseline	Day 7	Baseline	Day 7
Heart rate [bpm]	478.3 ± 50.5	423.2 ± 30.8	484.7 ± 64.6	466.2 ± 34.5
LVEDV [µl]	59.3 ± 7.3	45.1 ± 10.2*	63 ± 12.0	53 ± 9.4
LVESV [µl]	25.6 ± 4.7	27.3 ± 7.2	29.1 ± 5.9	29.9 ± 7.5
Stroke volume [µl]	33.8 ± 4.5	17.8 ± 4.4*	33.9 ± 7.3	23.1 ± 2.5*
Cardiac output [ml/min]	16.2 ± 3.1	7.6 ± 2.0*	15.2 ± 2.2	10.8 ± 1.6*
EF [%]	57.1 ± 4.8	39.9 ± 5.9*	53.9 ± 4.0	44.1 ± 4.8*
FS length [%]	16.6 ± 1.7	13.6 ± 1.5*	16.1 ± 2.0	16.2 ± 3.2
FAC [%]	55.1 ± 6.0	42.2 ± 7.5*	52.2 ± 6.4	45.2 ± 9.7
LVAWd [mm]	0.7 ± 0.09	0.9 ± 0.21*	0.7 ± 0.08	0.9 ± 0.17*
LVPWd [mm]	0.6 ± 0.09	0.7 ± 0.07	0.6 ± 0.06	0.6 ± 0.16
LVIDd [mm]	4.1 ± 0.15	3.5 ± 0.27*	4.2 ± 0.21	3.7 ± 0.36*
LV Mass [mg/g]	4 ± 0.5	5.2 ± 1.2*	3.9 ± 0.3	4.6 ± 0.5
MV E [mm/s]	848.8 ± 112.8	420.2 ± 92.8*	824.3 ± 88.3	546.3 ± 111.5*
MV A [mm/s]	568.5 ± 81.6	329.4 ± 71.3*	557.2 ± 111.9	377.7 ± 62.2*
MV E/A	1.5 ± 0.17	1.3 ± 0.23	1.5 ± 0.20	1.5 ± 0.28
IVRT [ms]	17 ± 2.8	23.6 ± 4.0*	15.8 ± 3.7	18.3 ± 6.0
IVCT [ms]	12 ± 2.0	16.2 ± 3.7*	13.3 ± 3.0	18.8 ± 2.3*
AET [ms]	48.3 ± 3.9	39.6 ± 3.3*	47.7 ± 4.7	45.1 ± 2.5
MV E Decel Time [ms]	24.4 ± 7.6	23.9 ± 3.2	20.9 ± 6.9	21 ± 3.1
Tei-Index	0.6 ± 0.08	1 ± 0.17*	0.6 ± 0.13	0.8 ± 0.20*
MV E' [mm/s]	23.9 ± 5.0	10.7 ± 4.2*	24.6 ± 5.0	15.5 ± 4.1*
MV A' [mm/s]	28.4 ± 4.9	16.8 ± 4.7*	30.2 ± 4.9	22.9 ± 3.7
MV E'/A'	0.9 ± 0.16	0.6 ± 0.19*	0.8 ± 0.08	0.7 ± 0.14
MV E/E'	36.3 ± 5.7	43.4 ± 13.8	34.2 ± 4.3	36.5 ± 8.1
S' [mm/s]	30.4 ± 6.3	23.2 ± 3.5*	29.1 ± 1.5	29.9 ± 4.0
GLS [%]	−20.2 ± 2.0	−14.3 ± 2.3*	−18.7 ± 1.7	−19.2 ± 2.4^#^

Male C57BL/6 J mice were infected with 10^5^ PFU CVB3 and treated with either sham (scrambled BK1.3-SCR control peptide), (*n* = 12) or BK1.3 (*n* = 12). Echocardiography was performed at baseline, 2 days prior to infection (sham: *n* = 12, BK1.3: *n* = 12), and on day 7 post-infection (sham: *n* = 8, BK1.3: *n* = 4). Echocardiographic parameters are presented as mean ± SD. Statistical analysis was conducted using a 2-way ANOVA followed by Tukey's test. Statistically significant results (*p* < 0.05) are indicated by * for comparisons between baseline and day 7 measurements within each treatment group, and by # for comparisons between the sham-treated and BK1.3-treated groups on day 7

LVEDV: Left Ventricular End-Diastolic Volume; LVESV: Left Ventricular End-Systolic Volume; LVEF: Left Ventricular Ejection Fraction; FS LV: Fractional Shortening of the Left Ventricle; FAC: Fractional Area Change; LVAWd: Left Ventricular Anterior Wall in Diastole; LVPWd: Left Ventricular Posterior Wall in Diastole; LVIDd: Left Ventricular Internal Diameter in Diastole; LV Mass: Left Ventricular Mass; MV: Mitral Valve; IVRT: Isovolumetric Relaxation Time; IVCT: Isovolumetric Contraction Time; AET: Aortic Ejection Time; MV E Decel: Mitral Valve E Deceleration; MV E Decel Time: Mitral Valve E Deceleration Time; GLS: Global longitudinal strain

## Discussion

This study aimed to evaluate the therapeutic potential of the chemokine-binding peptide BK1.3, derived from the broader-spectrum Evasin protein P672 in mitigating inflammation within a mouse model of viral myocarditis caused by Coxsackievirus B3 (CVB3). The findings demonstrate that BK1.3 effectively reduces cardiac inflammation, particularly by decreasing the infiltration of myeloid cells, without compromising virus control or exacerbating systemic pathology. These results align with the study’s primary goal of addressing the critical challenge in viral myocarditis: managing the detrimental inflammatory response without disrupting viral clearance or causing additional harm to the host.

Our research focused on the selective inhibition of key chemokines-CCL2, CCL3, CCL7, and CCL8-that are instrumental in the recruitment of pro-inflammatory myeloid cells to the myocardium. These chemokines have been extensively documented as central drivers of immune cell infiltration and subsequent inflammation in both human and experimental myocarditis models [3, 47]. By effectively targeting these chemokines, BK1.3 offers a promising therapeutic strategy to mitigate the excessive immune response that contributes to cardiac damage. The reduction in myeloid cell infiltration observed in BK1.3-treated animals directly correlates with the intended pharmacological action of the peptide, underscoring its specificity and potential efficacy.

The safety profile of BK1.3 and P672 was rigorously evaluated across multiple organ systems, focusing on both the acute (day 3) and subacute (day 8) phases of CVB3 infection. Systemic parameters, including body weight, glucose levels, and body temperature, were monitored to detect any signs of exacerbated systemic pathology. The absence of significant changes in these parameters among the treatment groups suggests that BK1.3 and P672 do not adversely affect general health during acute infection. Additionally, the lack of increased viral loads in key organs such as the pancreas and liver further indicates that these treatments do not impair the host's ability to control the viral infection, a critical aspect of safety for any immunomodulatory therapy.

Liver function, often compromised during systemic viral infections, was another focal point of our safety assessment. Despite the significant hepatic injury observed during the acute phase of CVB3 infection, BK1.3 treatment did not exacerbate liver damage. On the contrary, by day 8, liver enzyme levels had returned to near-normal levels in treated animals, indicating effective liver recovery. The lack of significant differences in histological scores for liver necrosis and inflammation between treated and control groups reinforces the conclusion that BK1.3 does not impede hepatic healing processes. This preservation of liver function, coupled with stable virus control, supports the safety and viability of BK1.3 as a therapeutic agent in viral myocarditis.

A particularly noteworthy finding is the selective impact of BK1.3 on myeloid cell infiltration into the myocardium. Previous studies have established the central role of myeloid cells, including monocytes and macrophages, in mediating the inflammatory response that exacerbates cardiac damage in myocarditis [3, 33]. The significant reduction in these cell populations following BK1.3 treatment aligns with the peptide’s mechanism of action, which involves the inhibition of chemokines specifically involved in myeloid cell recruitment. This selective inhibition is crucial, as it allows for the modulation of the inflammatory response without compromising the infiltration of lymphoid cells, which are essential for effective antiviral immunity.

Interestingly, T cell infiltration remained unaffected by BK1.3 treatment, despite the reduced viral load. This suggests that the initial viral burden detected on day 3 post-infection, which was comparable across all experimental groups, likely triggered both myeloid and lymphoid cell infiltration through similar concentrations of pathogen-associated molecular patterns (PAMPs). The fact that BK1.3 selectively reduced myeloid cell recruitment without affecting T-cell infiltration highlights its potential to modulate the immune response in a way that mitigates inflammation while preserving the essential components of antiviral defense.

The functional relevance of these cellular changes was confirmed through comprehensive echocardiographic assessments. The reduction in myeloid cell infiltration was accompanied by a marked improvement in left ventricular function in BK1.3-treated mice compared to sham-treated animals. Key echocardiographic parameters, including ejection fraction, cardiac output, and global longitudinal strain (GLS), showed significant improvement in BK1.3-treated animals compared to controls. GLS, in particular, is a sensitive marker of myocardial function, especially in conditions like myocarditis where focal lesions are common [3]. The preservation of cardiac function in BK1.3-treated animals underscores the therapeutic potential of this peptide in viral myocarditis, where excessive inflammation is a major contributor to disease progression.

Our findings align with existing literature that revealed the detrimental role of myeloid cells in the pathogenesis of viral myocarditis. Studies have shown that the infiltration of monocytes and macrophages into the myocardium exacerbates tissue damage, contributes to fibrosis, and impairs cardiac function [47]. The observed reduction in these cell populations with BK1.3 treatment is consistent with the hypothesis that targeted chemokine inhibition can mitigate these harmful effects without disrupting the broader immune response. The safety profile observed with BK1.3 treatment, including its lack of impact on virus control and systemic health, further supports its potential as a therapeutic option.

While this study provides compelling evidence for the efficacy and safety of BK1.3 in a preclinical model of viral myocarditis, several questions remain. Future research should explore the long-term effects of BK1.3 treatment on cardiac function and survival, particularly in the context of chronic myocarditis and heart failure. Additionally, the potential application of BK1.3 in other inflammatory cardiovascular diseases, where chemokine-mediated myeloid cell infiltration plays a critical role, warrants investigation. Finally, clinical studies are necessary to translate these findings into therapeutic strategies for patients suffering from viral myocarditis and other forms of inflammatory heart disease.

In summary, our study demonstrates that BK1.3 effectively reduces cardiac inflammation by selectively inhibiting the recruitment of pro-inflammatory myeloid cells, without compromising virus control or systemic health. These findings highlight the therapeutic potential of BK1.3 as a novel strategy for managing viral myocarditis and suggest that targeted chemokine inhibition could be a valuable approach in treating inflammatory heart diseases.

## Supplementary Information

Below is the link to the electronic supplementary material.

Supplementary file1 (PDF 6201 KB)

## Data Availability

The mass spectrometry proteomics data have been deposited to the ProteomeXchange Consortium via the PRIDE [51 partner repository with the dataset identifier PXD060461. All other data are available from the corresponding authors upon reasonable request.
