# Supplementary material for: Assessing customized multivalent chemokine-binding peptide treatment in a murine model of coxsackievirus B3 myocarditis
Source: Basic Res Cardiol. 2025 Feb 26;120(2):393–422. doi: 10.1007/s00395-025-01098-w (PMC11976344; doi:10.1007/s00395-025-01098-w)
Supplement: Supplementary file 1 — Supplementary file1 (PDF 6201 KB) [file 395_2025_1098_MOESM1_ESM.pdf]

SUPPLEMENTAL MATERIAL

Title

Assessing Customized Multivalent Chemokine-Binding Peptide Treatment in a Murine Model of Coxsackievirus B3 Myocarditis

Running title:
Evasin-derived peptide therapeutics in viral myocarditis

Journal

Basic Research in Cardiology

Authors

Nicolas Kelm[1], Meike Kespohl[1,2], Gintare Smagurauskaite[3], Serena Vales[3], Kalimuthu Karuppanan[3], Philomena Mburu[3], Arne Thiele[2,4-7], Sandra Pinkert[1], Thomas Bukur[8], Michael Mülleder[9], Nikolaus Berndt[10-12], Karin Klingel[13], Matthias M. Gaida[8,14,15], Shoumo Bhattacharya[3] and Antje Beling[1,2*]

1 – Charité – Universitätsmedizin Berlin, corporate member of Freie Universität Berlin and Humboldt-Universität zu Berlin, Institute of Biochemistry, 10117 Berlin, Germany
2 – Deutsches Zentrum für Herz-Kreislauf-Forschung, partner site Berlin, 10117 Berlin, Germany
3 – Centre for Human Genetics and RDM Cardiovascular Medicine, University of Oxford, Roosevelt Drive, Oxford, OX3 7BN, United Kingdom
4 - Max Rubner Center for Cardiovascular Metabolic Renal Research, Institute of Pharmacology, Charité - Universitätsmedizin Berlin, Berlin, Germany
5 - Experimental and Clinical Research Center, a joint cooperation of Max-Delbrück Center for Molecular Medicine and Charité - Universitätsmedizin Berlin, Berlin, Germany
6 - Department of Nephrology and Intensive Care Medicine, Charité - Universitätsmedizin Berlin, Berlin, Germany
7 - Max-Delbrück-Center for Molecular Medicine in the Helmholtz Association, Berlin, Germany
8 - TRON - Translational Oncology at the University Medical Center of the Johannes Gutenberg University Mainz, Germany
9 - Core Facility High-Throughput Mass Spectrometry, Charité-Universitätsmedizin Berlin, Freie Universität Berlin and Humboldt-Universität zu Berlin, Germany
10 - Deutsches Herzzentrum der Charité, Institute of Computer-Assisted Cardiovascular Medicine, Berlin, Germany
11 - Charité-Universitätsmedizin Berlin, corporate member of Freie Universität Berlin and Humboldt-Universität zu Berlin, Germany
12 - German Institute of Human Nutrition Potsdam-Rehbruecke, Department of Molecular Toxicology, Nuthetal, Germany
13 - Cardiopathology, Institute for Pathology and Neuropathology, University Hospital Tübingen, 72076 Tübingen, Germany
14 - Institute of Pathology, University Medical Center Mainz, Johannes-Gutenberg-Universität Mainz, 55131 Mainz, Germany
15 - Research Center for Immunotherapy, University Medical Center Mainz, Johannes-Gutenberg-Universität Mainz, 55131 Mainz, Germany

[*]**Corresponding author**

Prof. Antje Beling
Phone: 0049 30 450 528 187
Email: antje.beling@charite.de
Charité – Universitätsmedizin Berlin
Institute of Biochemistry
Charitéplatz 1
10117 Berlin
Germany

ORCID ID: 0000-0002-1826-5248

Supplemental figures

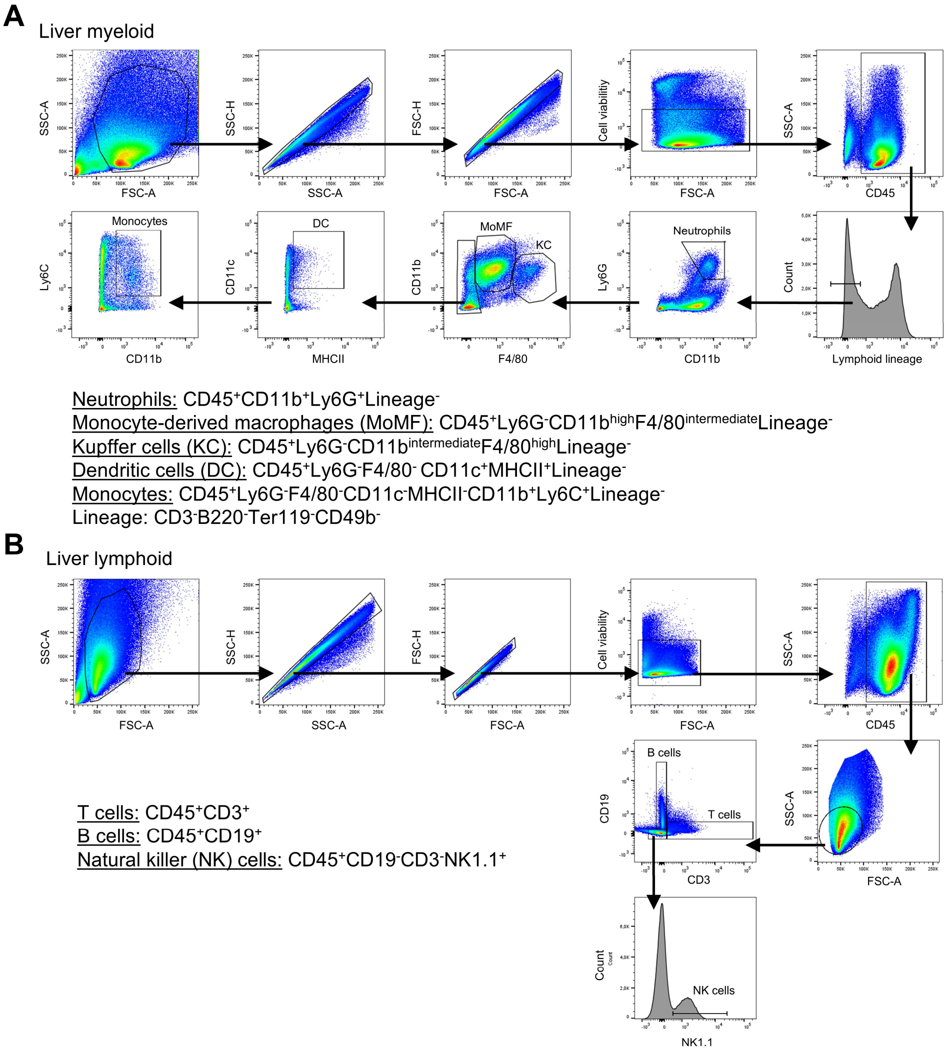

Figure S1: Applied gating strategy of flow cytometry analysis investigating hepatic immune cell infiltration. Immune cells were isolated from hepatic tissue, stained with fluorochrome-linked antibodies and measured with flow cytometry. Analysis of myeloid and lymphoid immune cells was done separately. The corresponding gating strategy is depicted for myeloid cells in (A) and for lymphoid cells in (B). In both analyses, cells were differentiated from debris by an FSC/SSC gate. Subsequently, cell doublets were eliminated by SSC-A/SSC-H and FSC-A/FSC-H gating. Staining with a fixable cell viability dye separated living cells from dead cells. Immune cell subpopulations were identified by combinations of cell surface markers as indicated. Immune cell marker-based differentiation of immune cell subpopulations adhered to current evidence [40].

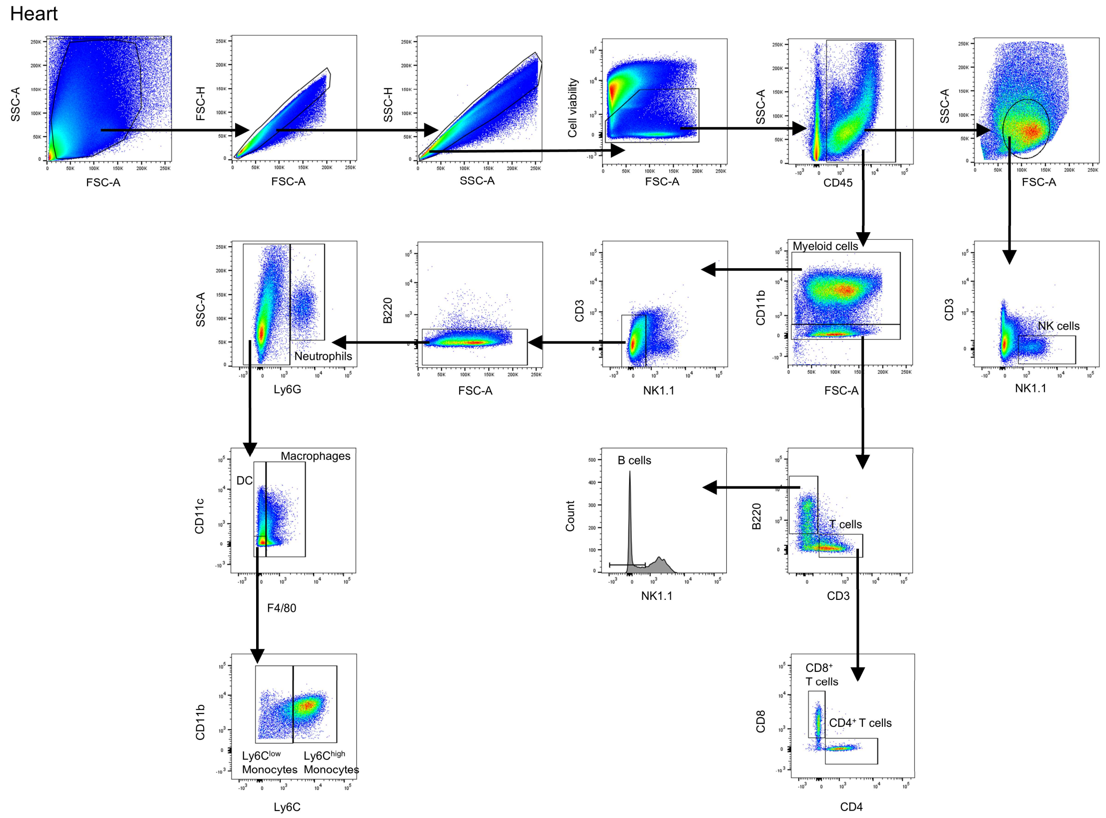

Myeloid cells: CD45$^+$CD11b$^+$
Neutrophils: CD45$^+$CD11b$^+$CD3$^-$NK1.1$^-$B220$^-$Ly6G$^+$
Macrophages: CD45$^+$CD11b$^+$CD3$^-$NK1.1$^-$B220$^-$Ly6G$^-$CD11c$^{+/-}$F4/80$^+$
Dendritic cells (DC): CD45$^+$CD11b$^+$CD3$^-$NK1.1$^-$B220$^-$Ly6G$^-$CD11c$^+$F4/80$^-$
Inflammatory monocytes: CD45$^+$CD11b$^+$CD3$^-$NK1.1$^-$B220$^-$Ly6G$^-$CD11c$^-$F4/80$^-$Ly6C^{high}
Patrolling monocytes: CD45$^+$CD11b$^+$CD3$^-$NK1.1$^-$B220$^-$Ly6G$^-$CD11c$^-$F4/80$^-$Ly6C^{low}
B cells: CD45$^+$CD11b$^-$B220$^+$CD3$^-$NK1.1$^-$
Natural killer (NK) cells: CD45$^+$CD3$^-$NK1.1$^+$
T cells: CD45$^+$CD11b$^-$B220$^-$CD3$^+$
CD4$^+$ T cells: CD45$^+$CD11b$^-$B220$^-$CD3$^+$CD4$^+$CD8$^-$
CD8$^+$ T cells: CD45$^+$CD11b$^-$B220$^-$CD3$^+$CD4$^-$CD8$^+$

Figure S2: Applied gating strategy of flow cytometry analysis investigating cardiac immune cell infiltration. Immune cells were isolated from cardiac tissue, stained with fluorochrome-linked antibodies and measured with flow cytometry. The corresponding gating strategy is depicted. Cells were differentiated from debris by an FSC/SSC gate. Subsequently, cell doublets were eliminated by SSC-A/SSC-H and FSC-A/FSC-H gating. Staining with a fixable cell viability dye separated living cells from dead cells. Immune cell subpopulations were identified by combinations of cell surface markers as indicated. The gating strategy is based on previous studies [50].

A Spleen myeloid

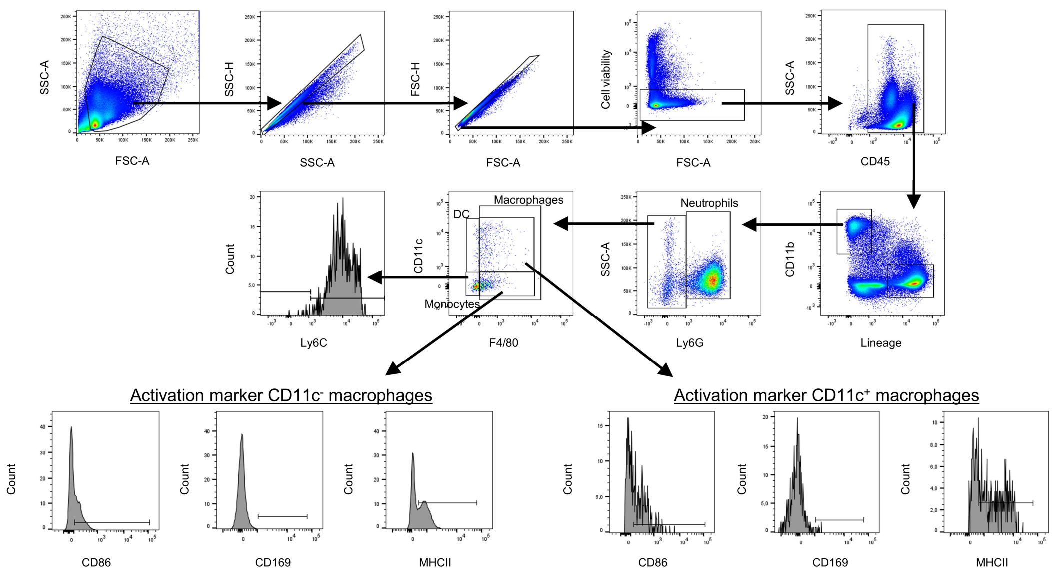

Neutrophils: CD45⁺CD11b⁺Ly6G⁺Lineage⁻
Dendritic cells (DCs): CD45⁺CD11b⁺Ly6G⁻CD11c⁺F4/80⁻Lineage⁻
Macrophages: CD45⁺CD11b⁺Ly6G⁻CD11c⁺/⁻F4/80⁺Lineage⁻
CD11c⁻ macrophages: CD45⁺CD11b⁺Ly6G⁻CD11c⁻F4/80⁺Lineage⁻
CD11c⁺ macrophages: CD45⁺CD11b⁺Ly6G⁻CD11c⁺F4/80⁺Lineage⁻
Ly6C⁺ monocytes: CD45⁺CD11b⁺Ly6G⁻CD11c⁻F4/80⁻Ly6C⁺Lineage⁻
Ly6C⁻ monocytes: CD45⁺CD11b⁺Ly6G⁻CD11c⁻F4/80⁻Ly6C⁻Lineage⁻

Activation marker for CD11c⁺/CD11c⁻ macrophages:
CD86⁺: % of CD11c⁺/CD11c⁻ macrophages
CD169⁺: % of CD11c⁺/CD11c⁻ macrophages
MHCII⁺: % of CD11c⁺/CD11c⁻ macrophages

Lineage: CD3⁻B220⁻Ter119⁻CD49b⁻

B Spleen lymphoid

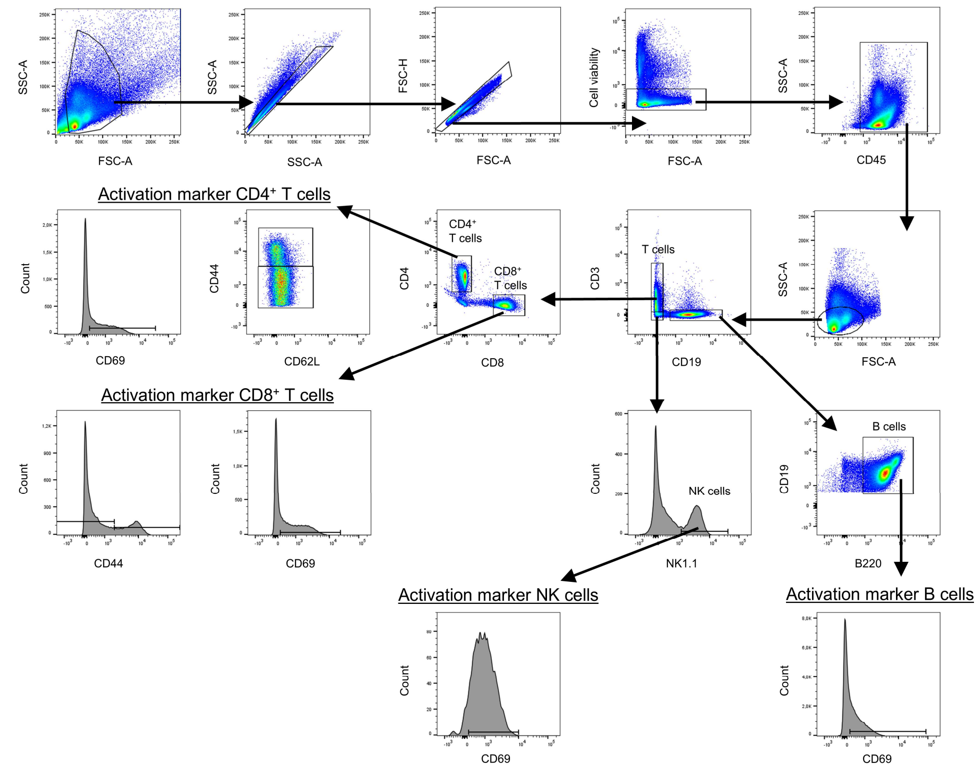

B cells: CD45⁺CD3⁻CD19⁺B220⁺
Natural killer (NK) cells: CD45⁺CD3⁻CD19⁻NK1.1⁺
T cells: CD45⁺CD19⁻CD3⁺
CD4⁺ T cells: CD45⁺CD19⁻CD3⁺CD4⁺CD8⁻
CD8⁺ T cells: CD45⁺CD19⁻CD3⁺CD4⁻CD8⁺

Activation marker for CD4⁺ T cells:
CD69⁺: % of CD4⁺ T cells
CD44^high: % of CD4⁺ T cells

Activation marker for CD8⁺ T cells:
CD69⁺: % of CD8⁺ T cells
CD44^high: % of CD8⁺ T cells

Activation marker for B cells:
CD69⁺: % of B cells

Activation marker for NK cells:
CD69⁺: % of NK cells

Figure S3: Applied gating strategy of flow cytometry analysis investigating immune cell mobilization and activation in splenic tissue. Immune cells were isolated from splenic tissue, stained with fluorochrome-linked antibodies and measured with flow cytometry. Analysis of myeloid and lymphoid immune cells was done separately. The corresponding gating strategy is depicted for myeloid cells in (A) and for lymphoid cells in (B). In both analyses, cells were differentiated from debris by an FSC/SSC gate. Subsequently, cell doublets were eliminated by SSC-A/SSC-H and FSC-A/FSC-H gating. Staining with a fixable cell viability dye separated living cells from dead cells. Immune cell subpopulations were identified by combinations of cell surface markers as indicated. Immune cell activation was assessed with activation markers using CD86, CD169 and MHCII for myeloid immune cells and CD69 and CD44 for lymphoid immune cells. The percentage of activation marker positive immune cells refers to the corresponding immune cell subset. The gating strategy is based on previous studies [50].

Supplemental tables

**Table S1: Details on the antibody fluorochrome conjugates used in flow cytometry experiments**				
**Antibodies used for flow cytometry of BALF**				
antibody - fluorochrome	clone	dilution	company	catalogue number
CD45 - PE	REA737	1:50	Miltenyi	130-119-798
Ly6G - PE-Vio770	REA526	1:50		130-107-915
Ly6C -APC	REA796	1:50		130-111-779
CD3 – FITC	REA641	1:50		130-119-798
**Antibodies used for flow cytometry of heart tissue**				
antibody - fluorochrome	clone	dilution	company	catalogue number
B220 - BUV395	RA3-6B2	1:200	BD	563793
CD8 - PB	53-6.7	1:100		558106
MHCII - FITC	AF6-120.1	1:150		553551
CD3 - BUV737	145-2C11	1:200		612771
CD11b - BV510	M1/70	1:300	Biolegend	101245
NK1.1 - PE	PK136	1:300		108707
CD11c - PE Dazzle	N418	1:200		117348
Ly6G - BV605	1A8	1:400		127639
F4/80 - APC	BM8	1:100		123116
CD4 - PerCPCy5.5	RM4-5	1:300		100539
CD45.2 - BV711	104	1:200		109847
Ly6C - PeCy7	HK1.4	1:400		128018
**Antibodies used for flow cytometry of spleen and liver tissue**				
antibody - fluorochrome	clone	dilution	company	catalogue number
CD8 - PB	53-6.7	1:100	BD	558106
CD4 - V500	RM4-5	1:100		560782
CD45 - BV711	104	1:300		563685
CD3 - BUV395	500A2	1:400		740221
MHCII – FITC	AF6-120.1	1:150		553551
CD86 - BUV395	GL1	1:200		564199
B220 – FITC	RA3-6B2	1:200	Biolegend	103206
CD19 - PeCy7	6D5	1:400		115519
NK1.1 - PE-TR	PK136	1:300		108748
CD69 – APC	H1.2F3	1:300		104513
CD62L - AF700	MEL-14	1:200		104426
CD169 - PerCPCy5.5	3D6.112	1:150		142409
CD3 - PE	145-2C11	1:300		100307
B220 – PE	RA3-6B2	1:300		103208
Ter119 – PE	TER-119	1:300		116208
CD49b – PE	DX5	1:300		108908
CD11c - PE-TR	N418	1:200		117348
Ly6C - PECy7	HK1.4	1:400		128018
F4/80 – APC	BM8	1:100		123116
CD45.2 - AF700	104	1:200		109822
CD11b - BV510	M1/70	1:300		101245
Ly6G - BV605	1A8	1:400		127639
CD44 - PE	IM7	1:400	Life Technologies	12-0441-81

Table S1: Details on the antibody fluorochrome conjugates used in flow cytometry experiments: The table S1 contains all antibody fluorochrome conjugates used in flow cytometry experiment to measure immune cell counts in heart, liver and spleen tissue. The

corresponding clones, the final dilutions within the master mix, the suppliers and catalogue numbers are listed.
